# Explaining Uphill Ion Transport in Multicomponent Solutions Across Membranes in Reverse Electrodialysis

**DOI:** 10.3390/membranes16080273

**Published:** 2026-08-16

**Authors:** Sigrid Aunsmo, Signe Kjelstrup, Odne S. Burheim, Simon B. B. Solberg

**Affiliations:** 1 PoreLab, Department of Chemistry and Biomedical Science, Norwegian University of Science and Technology, 7491 Trondheim, Norway; sigrid.aunsmo@ntnu.no (S.A.); signe.kjelstrup@ntnu.no (S.K.); 2 Department of Energy and Process Engineering, Norwegian University of Science and Technology, 7491 Trondheim, Norway; 3 Department of Engineering Science, University of Oxford Parks Road, Oxford OX1 3PJ, UK

**Keywords:** membrane transport number, multicomponent ion transport, uphill transport, membrane coupled transport, entropy production

## Abstract

Diffusion against a concentration gradient, also called uphill transport, has been reported for magnesium and sulfate ions in solutions of NaCl and Mg${\mathrm{SO}}_{4}$ in reverse electrodialysis (RED). Here we derive transport equations for such systems and explain the observed uphill transport using non-equilibrium thermodynamics (NET). A set of Nernst–Planck equations was reformulated into a set of flux equations with neutral salt driving forces. By applying the condition of entropy production invariance to the sets of variables, we show how an ideal ion selectivity model provides Onsager coupling coefficients for the ion transport. These coupling coefficients can predict and explain the observed uphill transport of magnesium and sulfate. A linear fitting scheme is developed to fit the transport coefficients to experimental data for the ideal ion selectivity model and for a more general model. The results show that even the simplest ideal ion selectivity model captures the uphill transport, and the phenomenon occurs due to diffusional ion exchange, as in Donnan dialysis. Under open-circuit conditions, deviations are large, and the simplest model is no longer sufficient; co-ion leakage corrections are essential. The results provide a basis for future modelling of RED processes; in particular, one can determine and optimise the process efficiency, as the model gives direct access to the process’s local entropy production. Osmosis is identified as a potential source of error.

## 1. Introduction

### 1.1. RED Background, Development and Uphill Transport Theory Gap

The idea to exploit the energy of mixing of salt and fresh water dates back to the 1950s [1,2,3,4]. One method for harvesting this energy is reverse electrodialysis (RED). The power of the RED stack stems from the controlled mixing of a concentrated electrolyte stream and a dilute one. A cation exchange membrane (CEM) and an anion exchange membrane (AEM) control this mixing and allow for the generation of an electric current. Development and implementation of the idea have been hampered, mainly because available membranes have been expensive and prone to the growth of biofilms [5,6]. Strategies to prevent membrane fouling have been developed [7,8,9,10], and efforts have been made to bring the technology to larger scales [11,12,13].

Efforts to improve other aspects of the RED performance have also been made, such as studies of the effect of hydraulic flow conditions [7,14,15,16], and the impact of essential membrane properties [17,18,19,20,21]. Increasing the overall temperature to decrease resistance [13,22], and imposing temperature gradients across the membranes [23] have been explored as ways to increase power output.

The most important variable, however, is the concentration of ionic species in the concentrate and dilute compartments used in the RED process, i.e., the main driving force for power generation. The availability of seawater makes it a natural choice for the concentrated stream in the RED process, but other sources that can provide larger concentration differences, such as brines, have also been considered [12,22,24,25,26]. Theoretical and experimental studies of RED mainly consider aqueous NaCl solutions. However, most relevant concentrate solutions, including seawater and brine, contain salts beyond NaCl. These salts are comprised of ionic species like ${\mathrm{Mg}}^{2+}$, K^+^, Ca^2+^, ${\mathrm{SO}}_{4}^{2-}$, Br^−^ and ${\mathrm{HCO}}_{3}^{-}$. The presence of these extra ions has been shown to impede the RED power output, primarily due to increased resistance in the ion exchange membranes [17,18,27,28,29,30,31,32]. It has also been observed that transport of ${\mathrm{Mg}}^{2+}$ and ${\mathrm{SO}}_{4}^{2-}$ ions across ion exchange membranes takes place against their concentration gradient if solutions contain three or more ionic species [32]. This phenomenon is often referred to as uphill transport, due to the transport of ions, such as ${\mathrm{Mg}}^{2+}$ and ${\mathrm{SO}}_{4}^{2-}$, from low to high concentration. It is this uphill transport and its thermodynamic description that we shall address in the present work.

Uphill transport has been observed both in the presence and absence of a net electric current in the RED system by Post et al. [32]. It was associated with a reduction in the stack voltage, an increase in the stack resistance, and consequently a reduction in stack power. It is of interest to understand why this phenomenon takes place. Post et al. noted, for open-circuit conditions, that the uphill transport in aqueous solutions with ${\mathrm{Na}}^{+}$, ${\mathrm{Cl}}^{-}$, ${\mathrm{Mg}}^{2+}$ and ${\mathrm{SO}}_{4}^{2-}$ mostly occurred as divalent ions in the dilute stream being exchanged for two monovalent ions in the concentrated stream, as in Donnan dialysis [32]. The electrochemical potential differences of the ions were proposed as driving forces for this exchange, but we shall see in this work that three driving forces of neutral salts are sufficient to explain the exchange. Some authors explain uphill transport by invoking local deviations from electroneutrality near the membrane [27], or even by deviations on the macro-scale in the concentrate and dilute streams [33]. Higa et al. pointed towards a membrane that preferentially binds divalent ions over monovalent ones, and an internal (downhill) concentration gradient driving transport of divalent ions uphill [34]. Moya conducted a numerical analysis of uphill transport using the Nernst–Planck equations for solutions of NaCl and MgCl_2_ separated by a cation exchange membrane [35]. They showed how the uphill transport can flip towards two ${\mathrm{Na}}^{+}$ being exchanged uphill for one ${\mathrm{Mg}}^{2+}$ transported downhill. The ratio of cation diffusion coefficients, or mobilities, was also stated as an important explanation for the phenomenon, and the ratio used in the study was $D_{{\mathrm{Na}}^{+}}=2D_{{\mathrm{Mg}}^{2+}}$. Other authors have also reported and discussed the uphill transport phenomenon [17,18,30,36,37,38,39,40]. Overall, there appears to be no clear consensus on either the mechanisms or the forces behind uphill transport in multicomponent electrochemical systems. A definite, controllable thermodynamic description can be said to be missing.

Uphill transport is generally acknowledged as a detrimental energy contribution to the RED process and its power output, but the exact link between uphill transport and the stack voltage reduction based on thermodynamics appears to be missing. In this work, we show how to explain the phenomenon already based on the Nernst–Planck equations, the corresponding ionic mobilities, and by the use of Onsager coupling as set up in the framework of non-equilibrium thermodynamics (NET). Usually, the Nernst–Planck equations are applied for all ions with the corresponding electrochemical potential gradients of ions as driving forces. We shall see that transforming the electrochemical potential gradients of ions into chemical potential gradients of neutral salts leads to a description more directly linked to experiments. The uphill transport phenomenon follows directly. The superposition of conduction and diffusion is an important aspect in that description.

Knowledge of these phenomena and their interaction will be essential for process optimisation and energy loss prevention. We shall shape the mass balances and transport equations into a form that is amenable to regression analysis, such that ion mobilities can be directly extracted from concentration data as a function of time. This method will be applied to the measurement data reported by Post et al. for the ${\mathrm{Na}}^{+}$, ${\mathrm{Cl}}^{-}$, ${\mathrm{Mg}}^{2+}$ and ${\mathrm{SO}}_{4}^{2-}$ system in a RED stack [32].

### 1.2. Outline

Our theoretical work is motivated by the experimental observations of Post et al., and these experiments will be used to verify our models. Therefore, we first include a section to briefly summarise the experiments of Post et al. and a description of how the experimental setup is simplified in our thermodynamical framework.

The derivation of the transport equations for the RED stack and how the assumption of ideal ion selectivity can be applied are presented in Section 3. The section further describes the linear fitting scheme used to fit transport coefficients to the experimental data. Finally, the section describes the particular case studies presented in this work.

The results in Section 4 present our fitted transport coefficients and show how well they are able to describe the RED stack. Coefficient fitting is performed with constraints imposed by the ideal ion selectivity assumption, leaving 4 independent unknown coefficients, and in a more general way with a relaxed model with 8 independent coefficients. Results will be presented for fits to both a standard-grade membrane RED stack and a monovalent-selective RED stack. Lastly, we provide a discussion in Section 5 about our model’s capability to describe these and other experiments, as well as aspects that could be taken into account for further modelling work.

## 2. Experimental Background

### 2.1. Description of Reported Results

Since the present study builds on experimental results readily reported in the literature, a description of the experiments conducted by Post et al. [32] is briefly provided here, followed by a description of the simplifications used in the model presented in this paper.

Post et al. connected four pairs of CEM and AEM (unit cells) in a RED flow cell stack, and the compartments between the membranes were formed by 0.5 mm silicone gaskets and spacers. Each electrolyte solution was fed into the stack at a rate of 57.5 mL min^−1^, leading to a residence time of roughly 0.5 min. Samples were taken at the stack outlet, and the solutions then flowed back into their respective containers in a batch process configuration. Only the concentration in the central, dilute compartment was measured. The area of the membranes was 0.01 $m^{2}$, and the volume of the solutions (container plus stack volume) was 1.05L.

The electric potential difference between the compartments housing the working and counter electrodes was measured with Ag|AgCl electrodes. One additional CEM was used in the stack to separate the electrode rinse solutions from the four unit cells. The measurements were carried out with initial concentrations of 3 mmol L^−1^ NaCl and 2 mmol L^−1^ Mg${\mathrm{SO}}_{4}$ in the dilute stream, and 450 mmol L^−1^ NaCl and 50 mmol L^−1^ Mg${\mathrm{SO}}_{4}$ in the concentrate stream. One batch experiment was conducted for open-circuit conditions, and another under current-producing conditions. In the latter scenario, an electric current was generated in cycles of 1000 s with a current density of 10 A m^−2^, or 100 mA, interspersed with 200 s of open-circuit conditions for open-circuit voltage (OCV) measurements.

Two separate RED stacks were used, both of them investigated under open-circuit and current-producing conditions. One stack contained standard-grade commercial ion exchange membranes (Neosepta CMX/AMX, Astom Co., Tokyo Japan., formerly Tokuyama Co.), while the other contained monovalent-selective membranes (Neosepta CMS/ACS, Astom Co., formerly Tokuyama Co.). The Neosepta CMS, with increased selectivity for monovalent ionic species, was prepared by coating a thin cationic polyelectrolyte layer on both sides of a standard-grade CMX membrane [32]. This layer of positive charge, on the otherwise negatively charged cation exchange membrane, provides a repulsive force that is stronger for divalent cations than monovalent ones. The Neosepta ACS was prepared by deposition of a cross-linked layer with small pores on both sides of an anion exchange membrane [32]. This layer provides a steric hindrance for larger molecules. Table 1 provides a summary of the membrane properties.

### 2.2. Model Considerations

**Figure 1:**
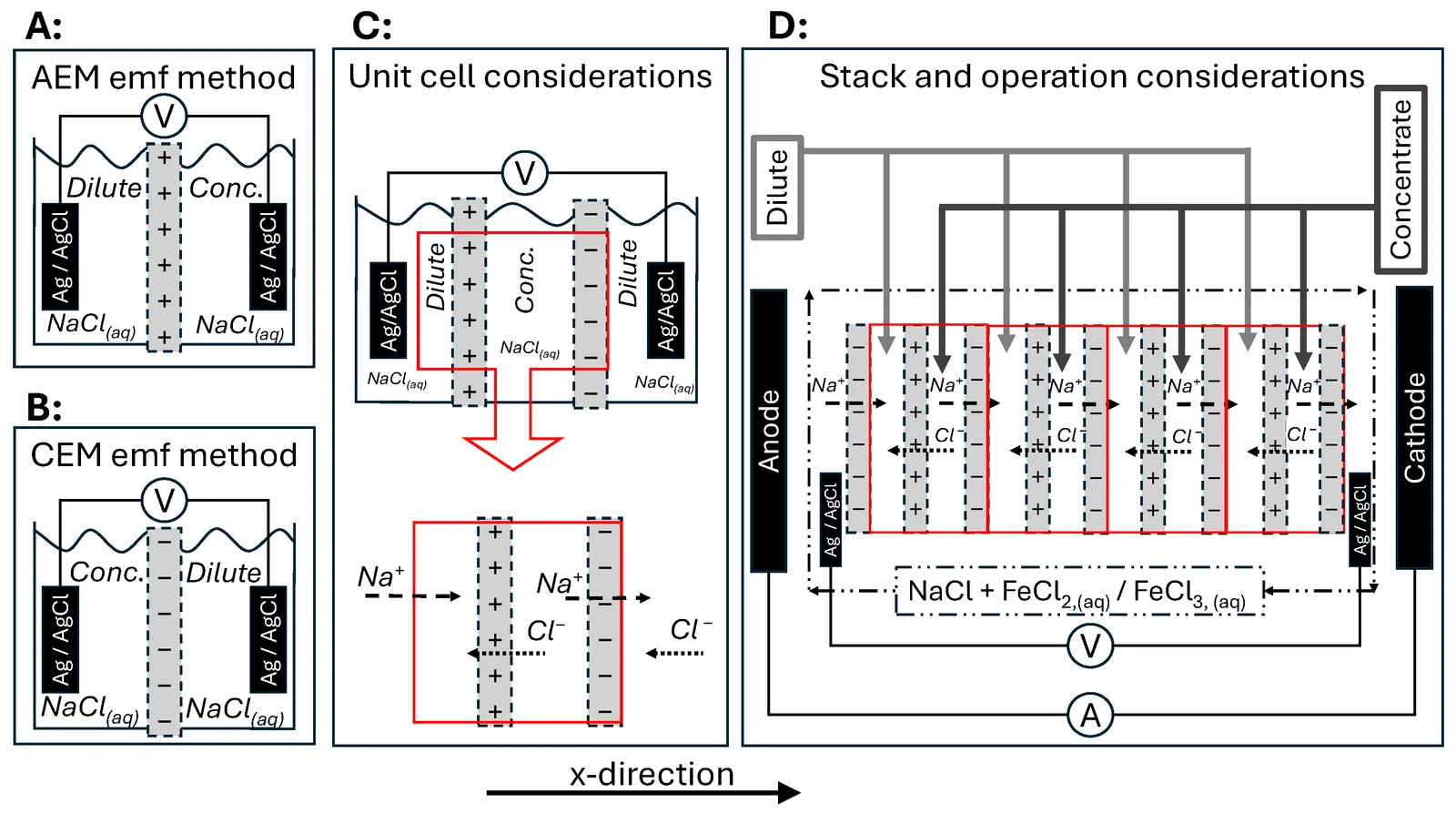
Relating membrane emf measurements between single membrane measurements (**A**,**B**) via unit cell consideration (**C**) to 4 stacked unit cells (**D**) in RED. The unit cells are marked in red and the arrows illustrate the cation and anion migration.

In order to derive our thermodynamical framework, we view the system setup in a simplified manner as illustrated in Figure 1C. One pair of a CEM and an AEM is what we refer to as a RED unit cell, and as shown in the Figure 1C, this is repeated 4 times in a RED stack. The chloride-reversible electrodes are placed in the outer chambers of the RED stack, and the open-circuit voltage is measured between the electrodes. In the literature, the inlets and outlets of the stack have been identified as points where an electric current can bypass pairs of membranes, i.e., the solution feeding the channels opens a channel for current leaks, in particular when too many (more than 6) unit cells are serially coupled. This leads directly to a loss in open-cell potential, and a lowering in coulombic and energy efficiency [44]. Since this setup has only four unit cells, we deem it reasonable to neglect this leakage current, and set the measured open-circuit voltage equal to the electromotive force in the transport equations. As illustrated in the figure, we refer to the measured open-circuit voltage as the emf, *V*.

The simplified RED stack model does not take into account that the solutions have a residence time in the chambers. It is rather assumed that each solution is well stirred, giving a uniform concentration within the chamber. The only relevant spatial direction is then the one perpendicular to the membranes; i.e., the *x*-direction (left to right) in Figure 1. In the description of the RED stack, the focus is not on the transport of ions from left to right, but rather on the fact that the transport of ions from concentrate to dilute solution is counted as positive. Section 3.4 will describe how to put together the CEM- and AEM-equations in order to model the RED stack.

Through the ion exchange membranes, water transport takes place and changes the solution volumes. Water transport has two main contributions: osmosis, where water is transported from high osmotic pressure to low osmotic pressure, and electro-osmosis, meaning that the ions that are transported through the membrane are accompanied by water molecules. In Appendix A we estimate that electro-osmosis would cause approximately a 2% volume increase in the dilute solution, whereas the worst-case estimate of osmosis would cause roughly a 9% volume decrease in the diluted solution. No measurements of volume change were made, so potentially modelled volume changes could not be validated. Therefore, we neglected the water transport altogether, keeping in mind that it can be included in theory and experiment when higher precision data are available. The central purpose of this work is to see how far we can model experimental data using ideal and relaxed ion selectivity models. We define the ideal ion selectivity assumption such that the ion exchange membrane allows only positive (CEM) or negative (AEM) counter-ions. In reality, there is always leakage of co-ions through a membrane, meaning that diffusion of anions in a CEM and cations in an AEM can take place. We shall see that the ideal ion selectivity model will dominate a description of the overall stack performance. We shall use the ideal ion-selective membrane model in order to gain insight into the effective transport equations and the origin of the uphill transport.

The central assumptions and simplifications that we employ in order to model the RED experiments may be summarised as follows:

- Contributions from electrode rinse solutions to voltage and transport are neglected.
- The four unit cells are identical, and solutions are well mixed.
- Any ions added to (removed from) the dilute stream are removed from (added to) the concentrate stream, allowing ion concentrations of the concentrate solution to be calculated from the known concentrations of the dilute solution.
- Hydrostatic pressure differences are negligible.
- Ionic shortcut currents [44] are negligible.
- The volume removed during sampling is negligible, and the total volume of each solution is constant.
- Electric current takes place between the two Ag|AgCl reference electrodes instead of the working/counter electrodes, a simplification that has no drawbacks since the total stack potential is not measured.
- No temperature was reported for the experiments; we assume it to be 25 °C.
- The water transport through the membranes is negligible.

We return to discuss the impact and validity of these assumptions in the discussion, Section 5.

## 3. Thermodynamical and Numerical Framework

This section has a theoretical part and a numerical part. In the theoretical part, we derive the effective transport equations for a RED stack using the theory of non-equilibrium thermodynamics (NET). This theory describes how ion fluxes depend on concentration gradients. The electric current and the emf of a RED stack are also connected. The theory requires the use of sets of independent variables, and we start by discussing two choices. The initial set consists of ion fluxes. Their driving forces follow from the entropy production. The second set of variables contains neutral electrolytes as variables. We find the fluxes and forces of this set using entropy production invariance. By including a second set of flux-force equations, we shall benefit from some direct relations between transport properties and experiments, not used before.

From the second variable set, using neutral salts as mass variables, we obtain a formulation where diffusion and conduction are superimposed. This makes it possible to combine equations for CEM (Figure 1B) and AEM (Figure 1A) into those of a unit cell (Figure 1C). The single membrane transport equations are finally combined into equations for the RED stack unit cell. Finally, we introduce the ideal ion selectivity model in its simplest form from the Nernst–Planck equations to see its impact on the effective RED unit cell description.

In the numerical part of the section, we develop a linear regression scheme. We linearise the time derivatives in the species mass balances and show how the equations can be restructured into a form that is amenable to linear regression. The regression coefficients that result are the transport properties of the ion exchange membranes. The experiments of Post and coworkers are used, providing new insight into the uphill-transport phenomenon described in the introduction [32].

### 3.1. Independent Component Variables in Salt Solutions of NaCl, Na*_2_*SO*_4_*, MgCl*_2_,* and MgSO*_4_*

The electrolyte solutions used in the RED stack experiment were prepared by dissolving NaCl and Mg${\mathrm{SO}}_{4}$ in water. After some time, ${\mathrm{Na}}^{+}$, ${\mathrm{Cl}}^{-}$, ${\mathrm{Mg}}^{2+}$ and ${\mathrm{SO}}_{4}^{2-}$ are present everywhere, in both the dilute and concentrate solution streams.

The four ion concentrations, $c_{i}$, form a natural set of mass variables. The four are not independent, however, as they are related by the electroneutrality condition:

(1) $$c_{{\mathrm{Na}}^{+}}+2c_{{\mathrm{Mg}}^{2+}}=c_{{\mathrm{Cl}}^{-}}+2c_{{\mathrm{SO}}_{4}^{2-}}.$$

In the presence of a net electric current, *j*, in the system, we need all four ion fluxes to be able to also describe the current:

(2) $$j/F=J_{{\mathrm{Na}}^{+}}-J_{{\mathrm{Cl}}^{-}}+2J_{{\mathrm{Mg}}^{2+}}-2J_{{\mathrm{SO}}_{4}^{2-}}.$$

Here, *F* is Faraday’s constant and $J_{i}$ is the molar flux of the ionic species *i*. The first natural set of flux-force variables is therefore (in the present case) the set of four ionic fluxes and their conjugate driving forces, the negative gradients in electrochemical potentials of the respective ions (see Section 3.2).

Electroneutrality can be violated on the microscale, near charged surfaces such as an ion exchange membrane or capacitive double layers (<10 nm). Energies of charge separation can, however, also be described by electroneutral compounds [45]. Consider therefore the chemical reaction equilibrium 2NaCl + MgSO_4_ ⇌ Na_2_SO_4_ + MgCl_2_. This relation between the four salts reduces the number of independent electrolyte components to three. The four chemical potentials in the chemical equilibrium are related by

(3) $$\mu_{{\mathrm{Na}}_{2}{\mathrm{SO}}_{4}}+\mu_{{\mathrm{MgCl}}_{2}}-2\mu_{\mathrm{NaCl}}-\mu_{{\mathrm{MgSO}}_{4}}=0,$$

where $\mu_{k}$ is the chemical potential of the neutral salt *k*. Three chemical potentials are independent and required to describe all possible composition and energy changes in the solution. We shall choose the components NaCl, MgSO_4_ and MgCl_2_. We may replace one of these three with Na_2_SO_4_, but prefer to use MgCl_2_ for reasons to become clear below. The fluxes of the three chosen salts are supplemented with the flux of charge, as measured in the external circuit. This means that there are four fluxes and correspondingly four driving forces in each of the two sets we are concerned with.

### 3.2. Entropy Production Invariance and Flux-Force Relations

The central insight from non-linear thermodynamics is that the entropy production, σ, is a sum of the products of independent fluxes $J_{k}$ and their conjugate forces $X_{k}$:

(4) $$\begin{array}{c} \sigma =\sum_{k}J_{k}X_{k}. \end{array}$$

In general, the forces are vectors in space; however, since the only relevant direction in the simplified RED system is perpendicular to the membranes, it is reduced to a one-dimensional problem. When discussing fluxes and forces, these would therefore always be considered to be in the *x*-direction as described in Figure 1. Further, the Onsager equations give the relation between the fluxes and forces through the Onsager coefficients, $L_{kj}$,

(5) $$\begin{array}{c} J_{k}=\sum_{j}L_{kj}X_{j}, \end{array}$$

for which Onsager proved the reciprocal relations $L_{kj}=L_{jk}$.

We discuss in more detail the two sets of flux-force equations from the previous section. The choice of variables has a bearing on the form of entropy production, its fluxes, and its conjugate driving forces. In the NaCl and MgSO_4_ aqueous solutions, there are four independent ion fluxes. An ion flux has—as a driving force—the electrochemical potential gradient of the ion, and the entropy production in an isothermal system can be written as follows:

(6) $$T\sigma =-J_{{\mathrm{Na}}^{+}}\partial_{x}{\tilde{\mu }}_{{\mathrm{Na}}^{+}}-J_{{\mathrm{Mg}}^{2+}}\partial_{x}{\tilde{\mu }}_{{\mathrm{Mg}}^{2+}}-J_{{\mathrm{Cl}}^{-}}\partial_{x}{\tilde{\mu }}_{{\mathrm{Cl}}^{-}}-J_{{\mathrm{SO}}_{4}^{2-}}\partial_{x}{\tilde{\mu }}_{{\mathrm{SO}}_{4}^{2-}}.$$

Here, *T* is the temperature of the isothermal system, ${\tilde{\mu }}_{i}$ is the electrochemical potential of ion *i*, and the notation $\partial_{x}$ is used for the unidirectional gradient, ∂/∂x. The electrochemical potential is defined as:

(7) $${\tilde{\mu }}_{i}=\mu_{i}+z_{i}\psi ,$$

where $\mu_{i}$ is the ion chemical potential that drives transport due to concentration gradients, $z_{i}$ is the charge number of the ion and ψ is the electrostatic potential that drives migration of ions. The ion fluxes have a membrane frame of reference, recalling that water transport is neglected in this work. If relevant, an extra term can be added to the entropy production. We summarise the local transport equations for the ion basis as

(8) $$\begin{array}{c} \overset{J^{\mathrm{ion}}}{\overset{︷}{\left[\begin{array}{c} J_{{\mathrm{Na}}^{+}}\\ J_{{\mathrm{Cl}}^{-}}\\ J_{{\mathrm{Mg}}^{2+}}\\ J_{{\mathrm{SO}}_{4}^{2-}} \end{array}\right]}}=\overset{L^{\mathrm{ion}}}{\overset{︷}{\left[\begin{array}{cccc} L_{11}^{\mathrm{ion}} & L_{12}^{\mathrm{ion}} & L_{13}^{\mathrm{ion}} & L_{14}^{\mathrm{ion}}\\ L_{21}^{\mathrm{ion}} & L_{22}^{\mathrm{ion}} & L_{23}^{\mathrm{ion}} & L_{24}^{\mathrm{ion}}\\ L_{31}^{\mathrm{ion}} & L_{32}^{\mathrm{ion}} & L_{33}^{\mathrm{ion}} & L_{34}^{\mathrm{ion}}\\ L_{41}^{\mathrm{ion}} & L_{42}^{\mathrm{ion}} & L_{43}^{\mathrm{ion}} & L_{44}^{\mathrm{ion}} \end{array}\right]}}\overset{X^{\mathrm{ion}}}{\overset{︷}{\frac{1}{T}\left[\begin{array}{c} -\partial_{x}{\tilde{\mu }}_{{\mathrm{Na}}^{+}}\\ -\partial_{x}{\tilde{\mu }}_{{\mathrm{Cl}}^{-}}\\ -\partial_{x}{\tilde{\mu }}_{{\mathrm{Mg}}^{2+}}\\ -\partial_{x}{\tilde{\mu }}_{{\mathrm{SO}}_{4}^{2-}} \end{array}\right]}}. \end{array}$$

A variable set that contains the neutral electrolytes is less intuitive, but also well established in the literature [3,46]. The essential part is that whichever choice we make, the flux-force products of the entropy production describe the same dissipation of energy. We utilize the electric current, Equation (2), to replace the chloride flux in Equation (6). This gives the following expression for the entropy production

(9) $$T\sigma =-J_{{\mathrm{Na}}^{+}}\partial_{x}\mu_{\mathrm{NaCl}}-J_{{\mathrm{Mg}}^{2+}}\partial_{x}\mu_{{\mathrm{MgCl}}_{2}}-J_{{\mathrm{SO}}_{4}^{2-}}\left(\partial_{x}\mu_{{\mathrm{MgSO}}_{4}}-\partial_{x}\mu_{{\mathrm{MgCl}}_{2}}\right)+j\partial_{x}{\tilde{\mu }}_{{\mathrm{Cl}}^{-}}/F.$$

Since chloride-reversible electrodes are used, we can identify the measurable electric potential (i.e., the driving force for net electric current) as $\partial_{x}{\tilde{\mu }}_{{\mathrm{Cl}}^{-}}=-F\partial_{x}\phi$ [3,46]. We can thus summarise the transport equations in this basis as

(10) $$\begin{array}{c} \overset{J}{\overset{︷}{\left[\begin{array}{c} J_{{\mathrm{Na}}^{+}}\\ J_{{\mathrm{Mg}}^{2+}}\\ J_{{\mathrm{SO}}_{4}^{2-}}\\ j/F \end{array}\right]}}=\overset{L}{\overset{︷}{\left[\begin{array}{cccc} L_{11} & L_{12} & L_{13} & L_{14}\\ L_{21} & L_{22} & L_{23} & L_{24}\\ L_{31} & L_{32} & L_{33} & L_{34}\\ L_{41} & L_{42} & L_{43} & L_{44} \end{array}\right]}}\overset{X}{\overset{︷}{\frac{1}{T}\left[\begin{array}{c} -\partial_{x}\mu_{\mathrm{NaCl}}\\ -\partial_{x}\mu_{{\mathrm{MgCl}}_{2}}\\ -\partial_{x}\left(\mu_{{\mathrm{MgSO}}_{4}}-\mu_{{\mathrm{MgCl}}_{2}}\right)\\ -F\partial_{x}\phi \end{array}\right]}}. \end{array}$$

To simplify notation, we write this flux and force vector as well as the Onsager coefficients without superscripts. We continue the derivations using the latter representation. However, we return to the ion representation when discussing the ideal ion selectivity assumption. To further ease the notation, we also introduce the subscripts 1 to 3 for the ion and salt fluxes and forces:

(11) $$\begin{array}{cc} J & =\left[\begin{array}{c} J_{{\mathrm{Na}}^{+}}\\ J_{{\mathrm{Mg}}^{2+}}\\ J_{{\mathrm{SO}}_{4}^{2-}}\\ j/F \end{array}\right]=\left[\begin{array}{c} J_{1}\\ J_{2}\\ J_{3}\\ j/F \end{array}\right],\;X=\frac{1}{T}\left[\begin{array}{c} -\partial_{x}\mu_{\mathrm{NaCl}}\\ -\partial_{x}\mu_{{\mathrm{MgCl}}_{2}}\\ -\partial_{x}\left(\mu_{{\mathrm{MgSO}}_{4}}-\mu_{{\mathrm{MgCl}}_{2}}\right)\\ -F\partial_{x}\phi \end{array}\right]=\left[\begin{array}{c} X_{1}\\ X_{2}\\ X_{3}\\ X_{\phi } \end{array}\right]. \end{array}$$

### 3.3. Transport Equations with Electroneutral Components

The local transport equations for the neutral components have now been established; however, a rearrangement is needed before putting together the unit cell equations. From the neutral salt flux-force equations in Equation (10), one cannot retrieve $X_{\phi }$ and $J_{i}$ from the experimental data (these are not measured for separate membranes but for the entire RED stack). Therefore, we choose to convert to using *j* and $X_{i}$ as input variables. These variables are obtainable for each membrane. In the last line of the flux-force, Equation (10), we exchange $X_{\phi }$ with *j* on the right-hand side of the equation,

(12) $$\begin{array}{cc} j/F & =L_{41}X_{1}+L_{42}X_{2}+L_{43}X_{3}+L_{44}X_{\phi }, \end{array}$$

(13) $$\begin{array}{cc} X_{\phi } & =\frac{1}{L_{44}}j/F-\frac{L_{41}}{L_{44}}X_{1}-\frac{L_{42}}{L_{44}}X_{2}-\frac{L_{43}}{L_{44}}X_{3}. \end{array}$$

This expression of $X_{\phi }$ is now introduced into the equations for $J_{1}$, $J_{2}$ and $J_{3}$, leading to a form suitable for describing the experimental results. The rearranged transport equations are:

(14) $$\begin{array}{c} \left[\begin{array}{c} J_{1}\\ J_{2}\\ J_{3}\\ X_{\phi } \end{array}\right]=\left[\begin{array}{cccc} \ell_{11} & \ell_{12} & \ell_{13} & t_{1}\\ \ell_{21} & \ell_{12} & \ell_{23} & t_{2}\\ \ell_{31} & \ell_{32} & \ell_{33} & t_{3}\\ -t_{1} & -t_{2} & -t_{3} & \frac{1}{\kappa } \end{array}\right]\left[\begin{array}{c} X_{1}\\ X_{2}\\ X_{3}\\ j/F \end{array}\right], \end{array}$$

where $L_{44}$ equals the electric conductivity, κ. The elements $\ell_{ij}$ in the matrix are related to the phenomenological coefficients $L_{ij}$ by

(15) $$\begin{array}{c} \ell_{ij}=L_{ij}-\frac{L_{i4}L_{4j}}{L_{44}}=L_{ij}-t_{i}t_{j}L_{44}, \end{array}$$

so that the $\ell_{ij}$ coefficients can be expressed by the transference coefficients. Lumped transference coefficients, $t_{1,2,3}$, have here been introduced in the coefficient matrix as

(16) $$t_{i}={\left(\frac{J_{i}}{j/F}\right)}_{d\mu_{i}=0}=\frac{L_{i4}}{L_{44}},\;\left\{t_{1}=\tau_{{\mathrm{Na}}^{+}},\;t_{2}=\frac{\tau_{{\mathrm{Mg}}^{2+}}}{2},\;t_{3}=-\frac{\tau_{{\mathrm{SO}}_{4}^{2-}}}{2}\right\},$$

with their relation to the ionic transport numbers, $\tau_{i}$, shown in braces. Transference coefficients and transport numbers are often confused. We clarify what is meant by the terms as follows:

- *Transference coefficient*, also called apparent transference number, $t_{i}=\frac{J_{i}}{j/F}{|}_{\Delta \mu =0}$, is the ratio of the amount of salt transferred between two solutions to the electric current in the external circuit.
- *Transport number *of an ion, $\tau_{i}=\frac{z_{i}J_{i}}{j/F}{|}_{\Delta \mu =0}$, describes the fraction of the total electric current that is carried by that particular ion. Transport numbers are often symbolised by $t_{i}$, but for clarity we use $\tau_{i}$ in this manuscript. The transport numbers sum up to one as the total electric current in the solution is carried by ions, $\sum_{i}\tau_{i}=1$.

The form of the flux-force relations given by Equation (14) is a convenient form because it contains a clear separation of phenomena: into a description of pure diffusion (the $\ell_{ij}$-matrix of coefficients) and into charge transfer (described by $t_{i}$). The same decomposition is characteristic in descriptions of Hittorf-type measurements [46]. It expresses that the effect of pure diffusion is superimposed on charge transfer. Diffusion alone is described when we introduce the condition j=0 [45,46]. The driving forces of Equation (14) are all gradients. They can be related to experimental values after integration. We have assumed the coefficients to be constant. Integration across the membrane is not possible, as we do not know the membrane composition. Following Scatchard (see [46]), we integrate instead over a series of aqueous solutions in equilibrium with the membrane. Using the assumption of local equilibrium, we thus obtain the forces of CEM and AEM in discrete form, to be used in the next sections.

### 3.4. Transport Equations of the Unit Cell

The transport coefficients, the *ℓ* matrix, will be different for the CEM and the AEM. We will now see how to combine flux and force relations for the CEM and AEM into effective equations for a RED unit cell. To describe the behaviour of the RED system, the relevant ion fluxes are those from the concentrate to the dilute compartment, rather than those from left to right. We will refer to the fluxes as the effective ion fluxes for a unit cell. From Figure 1, we see that these fluxes are in the positive *x*-direction through the CEM and in the negative *x*-direction through the AEM. Thus, the effective ion flux from concentrate to dilute will be $J_{i}^{*}=J_{i}^{\mathrm{CEM}}-J_{i}^{\mathrm{AEM}}$, where we have introduced a superscript to mark the contributions from the membranes and a star superscript to denote the effective flux. The electric current, however, has the same direction through both membranes. Under the assumption of equal membrane thickness and uniform concentration within the solutions, it is clear that the chemical potential gradients are opposite for the CEM and the AEM, $X_{1,2,3}^{\mathrm{AEM}}=-X_{1,2,3}^{\mathrm{CEM}}$. For the voltage, the measurable quantity will be the sum of the voltage over the CEM and AEM. The effective fluxes and forces for a RED unit cell are summarised as follows:

(17) $$\begin{array}{c} \left[\begin{array}{c} J_{1}^{*}\\ J_{2}^{*}\\ J_{3}^{*}\\ X_{\phi }^{*} \end{array}\right]\equiv \left[\begin{array}{c} J_{1}^{\mathrm{CEM}}-J_{1}^{\mathrm{AEM}}\\ J_{2}^{\mathrm{CEM}}-J_{2}^{\mathrm{AEM}}\\ J_{3}^{\mathrm{CEM}}-J_{3}^{\mathrm{AEM}}\\ X_{\phi }^{\mathrm{CEM}}+X_{\phi }^{\mathrm{AEM}} \end{array}\right],\;\left[\begin{array}{c} X_{1}^{*}\\ X_{2}^{*}\\ X_{3}^{*}\\ j/F \end{array}\right]\equiv \left[\begin{array}{c} X_{1}^{\mathrm{CEM}}\\ X_{2}^{\mathrm{CEM}}\\ X_{3}^{\mathrm{CEM}}\\ j/F \end{array}\right]=\left[\begin{array}{c} -X_{1}^{\mathrm{AEM}}\\ -X_{2}^{\mathrm{AEM}}\\ -X_{3}^{\mathrm{AEM}}\\ j/F \end{array}\right]. \end{array}$$

From conservation of mass, the effective flux can be used to express the time rate of change of the concentration of the component in question. The chloride concentration can be calculated from the other ions by enforcing electroneutrality. By combining the single membrane transport equations from Equation (14), the effective unit cell transport equations are found:

(18) $$\begin{array}{cc} \left[\begin{array}{c} J_{1}^{*}\\ J_{2}^{*}\\ J_{3}^{*}\\ X_{\phi }^{*} \end{array}\right] & =\overset{\ell^{*}}{\overset{︷}{\left[\begin{array}{cccc} \left(\ell_{11}^{\mathrm{CEM}}+\ell_{11}^{\mathrm{AEM}}\right) & \left(\ell_{12}^{\mathrm{CEM}}+\ell_{12}^{\mathrm{AEM}}\right) & \left(\ell_{13}^{\mathrm{CEM}}+\ell_{13}^{\mathrm{AEM}}\right) & \left(t_{1}^{\mathrm{CEM}}-t_{1}^{\mathrm{AEM}}\right)\\ \left(\ell_{21}^{\mathrm{CEM}}+\ell_{21}^{\mathrm{AEM}}\right) & \left(\ell_{22}^{\mathrm{CEM}}+\ell_{22}^{\mathrm{AEM}}\right) & \left(\ell_{23}^{\mathrm{CEM}}+\ell_{23}^{\mathrm{AEM}}\right) & \left(t_{2}^{\mathrm{CEM}}-t_{2}^{\mathrm{AEM}}\right)\\ \left(\ell_{31}^{\mathrm{CEM}}+\ell_{31}^{\mathrm{AEM}}\right) & \left(\ell_{32}^{\mathrm{CEM}}+\ell_{32}^{\mathrm{AEM}}\right) & \left(\ell_{33}^{\mathrm{CEM}}+\ell_{33}^{\mathrm{AEM}}\right) & \left(t_{3}^{\mathrm{CEM}}-t_{3}^{\mathrm{AEM}}\right)\\ -(t_{1}^{\mathrm{CEM}}-t_{1}^{\mathrm{AEM}}) & -(t_{2}^{\mathrm{CEM}}-t_{2}^{\mathrm{AEM}}) & -(t_{3}^{\mathrm{CEM}}-t_{3}^{\mathrm{AEM}}) & \left(\frac{1}{\kappa^{\mathrm{CEM}}}+\frac{1}{\kappa^{\mathrm{AEM}}}\right) \end{array}\right]}}\left[\begin{array}{c} X_{1}^{\mathrm{CEM}}\\ X_{2}^{\mathrm{CEM}}\\ X_{3}^{\mathrm{CEM}}\\ j^{\mathrm{CEM}}/F \end{array}\right]. \end{array}$$

Notice that effective transference numbers are subtracted whereas the other coefficients are added for the two types of membranes. It is, in principle, these effective coefficients that we can determine through parameter regression. However, we shall see in the next section that under the ideal ion selectivity assumption, estimates can also be made for the single membrane coefficients.

### 3.5. The Nernst–Planck Equations and the Ideal Ion Selectivity Assumption

So far, all transport equations have been general. No assumptions have been made about membrane properties. In the next step, we apply simplifying assumptions. It is very common to assume that the flux of an ion is proportional to the gradient of the electrochemical potential of that ion only. The Nernst–Planck equations arise from this assumption and can be expressed in their simplest form with ion mobilities $u_{i}$ as:

(19) $$\begin{array}{c} J_{i}=-\frac{c_{i}u_{i}}{Fz_{i}}\partial_{x}{\tilde{\mu }}_{i}. \end{array}$$

For a system of four ions, the set of Nernst–Planck equations takes the form:

(20) $$\begin{array}{c} J^{\mathrm{ion}}=\left[\begin{array}{cccc} U_{{\mathrm{Na}}^{+}} & 0 & 0 & 0\\ 0 & U_{{\mathrm{Cl}}^{-}} & 0 & 0\\ 0 & 0 & U_{{\mathrm{Mg}}^{2+}} & 0\\ 0 & 0 & 0 & U_{{\mathrm{SO}}_{4}^{2-}} \end{array}\right]X^{\mathrm{ion}} \end{array}$$

where we introduce $U_{i}$, which we refer to as total ion mobility:

(21) $$\begin{array}{c} U_{i}\equiv \frac{c_{i}u_{i}}{Fz_{i}}. \end{array}$$

In this work we shall determine the total ion mobilities, $U_{i}$, which are assumed to be constant in the concentration ranges considered. We connect this model to the experimental results, using effective transport equations. By matrix transformation (see Appendix B), we first find the matrix of transport coefficients in the neutral electrolyte representation. It is equal to

(22) $$\begin{array}{cc} L\; & =\left[\begin{array}{cccc} U_{{\mathrm{Na}}^{+}} & 0 & 0 & U_{{\mathrm{Na}}^{+}}\\ 0 & U_{{\mathrm{Mg}}^{2+}} & 0 & 2U_{{\mathrm{Mg}}^{2+}}\\ 0 & 0 & U_{{\mathrm{SO}}_{4}^{2-}} & -2U_{{\mathrm{SO}}_{4}^{2-}}\\ U_{{\mathrm{Na}}^{+}} & 2U_{{\mathrm{Mg}}^{2+}} & -2U_{{\mathrm{SO}}_{4}^{2-}} & \kappa \end{array}\right], \end{array}$$

The electric conductivity of the membrane, κ, is then the sum of contributions from ions $\kappa =U_{{\mathrm{Na}}^{+}}+U_{{\mathrm{Cl}}^{-}}+4U_{{\mathrm{Mg}}^{2+}}+4U_{{\mathrm{SO}}_{4}^{2-}}$, and the transport number of an ion is the fraction of the electric current carried by ion *i*:

(23) $$\tau_{i}\equiv {\left(\frac{z_{i}c_{i}u_{i}}{\sum z_{j}c_{j}u_{j}}\right)}_{dc_{j}=0}=\frac{z_{i}^{2}U_{i}}{\kappa },$$

where $U_{i}$ is adapted to the components, species and concentrations applied in this study. At the outset, there are 10 unknowns in the coefficient matrix. By using the Nernst–Planck equations for uncoupled ion transport, this was reduced to four unknowns. The ideal ion selectivity model is applied to each membrane. Only cations are then allowed to pass through the CEM, and only anions are allowed through the AEM. This is equivalent to saying that the mobilities of anions through the cation exchange membrane are zero $u_{{\mathrm{Cl}}^{-}}^{\mathrm{CEM}}=u_{{\mathrm{SO}}_{4}^{2-}}^{\mathrm{CEM}}=0$ and that the cation mobilities in the anion membrane are zero, $u_{{\mathrm{Na}}^{+}}^{\mathrm{AEM}}=u_{{\mathrm{Mg}}^{2+}}^{\mathrm{AEM}}=0$. The $\ell^{\mathrm{CEM}}$ and $\ell^{\mathrm{AEM}}$ matrices are found using Equations (15) and (16). From Equation (18), the effective unit cell transport matrix becomes

(24) $$\begin{array}{cc} \ell^{*,\mathrm{ideal}} & =\left[\begin{array}{cccc} 4\left(\frac{U_{{\mathrm{Na}}^{+}}U_{{\mathrm{Mg}}^{2+}}}{\kappa^{\mathrm{CEM}}}\right) & -2\left(\frac{U_{{\mathrm{Na}}^{+}}U_{{\mathrm{Mg}}^{2+}}}{\kappa^{\mathrm{CEM}}}\right) & 0 & \frac{U_{{\mathrm{Na}}^{+}}}{\kappa^{\mathrm{CEM}}}\\ -2\left(\frac{U_{{\mathrm{Na}}^{+}}U_{{\mathrm{Mg}}^{2+}}}{\kappa^{\mathrm{CEM}}}\right) & \left(\frac{U_{{\mathrm{Na}}^{+}}U_{{\mathrm{Mg}}^{2+}}}{\kappa^{\mathrm{CEM}}}\right) & 0 & \frac{1}{2}\left(1-\frac{U_{{\mathrm{Na}}^{+}}}{\kappa^{\mathrm{CEM}}}\right)\\ 0 & 0 & \left(\frac{U_{{\mathrm{SO}}_{4}^{2-}}U_{{\mathrm{Cl}}^{-}}}{\kappa^{\mathrm{AEM}}}\right) & \frac{2U_{{\mathrm{SO}}_{4}^{2-}}}{\kappa^{\mathrm{AEM}}}\\ -\frac{U_{{\mathrm{Na}}^{+}}}{\kappa^{\mathrm{CEM}}} & -\frac{1}{2}\left(1-\frac{U_{{\mathrm{Na}}^{+}}}{\kappa^{\mathrm{CEM}}}\right) & -\frac{2U_{{\mathrm{SO}}_{4}^{2-}}}{\kappa^{\mathrm{AEM}}} & \frac{1}{\kappa^{\mathrm{CEM}}}+\frac{1}{\kappa^{\mathrm{AEM}}} \end{array}\right]. \end{array}$$

This is the form of the $\ell^{*}$-matrix that we refer to as the ideal ion selectivity model. We notice several characteristic properties. Each of the membranes, CEM or AEM, is described by two ion mobilities alone. The upper left corner describes diffusion (the terms on the diagonal) and interdiffusion (the coupling terms) for Na^+^ and Mg^2+^ in CEM. The third column and row arise from ion transport in the AEM through the anion selectivity. In both membranes, ions can interchange proportionally according to their ionic charges, 2:1. The coupling coefficients that describe interdiffusion, $\ell_{21}^{*}=\ell_{12}^{*}$, are therefore non-zero. But $\ell_{13}^{*}$ and $\ell_{23}^{*}$, which can be connected to membrane leaks (imperfectness), are zero here. The coupling coefficient $\ell_{12}^{*}$ is negative, because it describes the interchange of ions in an electroneutral environment. We shall see that these coefficients are essential in a description of uphill transport of Mg^2+^ ions. They also turn out to be sufficient for a description of this transport. From the elements of the $\ell^{*}$-matrix, we shall find the mobilities of the ideal ion-selective model, separately for CEM and AEM.

### 3.6. Numerical Calculations

The experimental data contain time series of measured ion concentrations and emf, which we use to regress transport coefficients. The first point of the concentration curve (at t=0) has the highest uncertainty in the time of the measurement, and was thus omitted in the fitting.

The driving forces $X_{1,2,3}$ were computed from the salt concentrations of the dilute and concentrate solutions. In the calculations of the driving forces, we assumed that the activity coefficients were constant in the concentration range investigated. We thus approximated the forces as follows:

(25) $$\begin{array}{cc} X_{1} & =\frac{R}{d}\ln\left(\frac{c_{{\mathrm{Na}}^{+}}^{C}c_{{\mathrm{Cl}}^{-}}^{C}}{c_{{\mathrm{Na}}^{+}}^{D}c_{{\mathrm{Cl}}^{-}}^{D}}\right), \end{array}$$

(26) $$\begin{array}{cc} X_{2} & =\frac{R}{d}\ln\left(\frac{c_{{\mathrm{Mg}}^{2+}}^{C}{\left(c_{{\mathrm{Cl}}^{-}}^{C}\right)}^{2}}{c_{{\mathrm{Mg}}^{2+}}^{D}{\left(c_{{\mathrm{Cl}}^{-}}^{D}\right)}^{2}}\right),\;X_{3}=\frac{R}{d}\ln\left(\frac{c_{{\mathrm{SO}}_{4}^{2-}}^{C}{\left(c_{{\mathrm{Cl}}^{-}}^{D}\right)}^{2}}{c_{{\mathrm{SO}}_{4}^{2-}}^{D}{\left(c_{{\mathrm{Cl}}^{-}}^{C}\right)}^{2}}\right), \end{array}$$

where *d* is the membrane thickness, *R* is the gas constant and $c_{i}$ is the concentration of ion *i* in the compartment, indicated by superscript C for concentrate and D for dilute. We further assume all membranes to have the same thickness, d=0.15 mm. The measured ion concentrations were used to calculate the forces. We did this to linearise the regression model.

The emf, or the open-circuit voltage, is expressed by

(27) $$\begin{array}{c} V_{j=0}=-\frac{4X_{\phi }^{*}Td}{F} \end{array}$$

where we defined $X_{\phi }^{*}$ to be the reversible contribution to the cell voltage, which we realised by setting κ=0 in $\ell^{*}$. The emf, as described by Equation (27), is then the open-circuit potential one expects to measure from a thermodynamic point of view.

Furthermore, with four unit cells in the RED stack, the time evolution of the concentration in the dilute compartment is described by

(28) $$\begin{array}{c} \partial_{t}c_{i}^{D}=\frac{4a}{V}J_{i}^{*}, \end{array}$$

where *a* is the area of one membrane and V is the volume of the dilute solution that was circulated through the dilute chambers of the RED stack. Altogether, the equations to be used can be summarised as follows:

(29) $$\begin{array}{c} \left[\begin{array}{c} \partial_{t}c_{1}^{D}\\ \partial_{t}c_{2}^{D}\\ \partial_{t}c_{3}^{D}\\ V_{j=0} \end{array}\right]=\left[\begin{array}{c} \frac{4a}{V}J_{1}^{*}\\ \frac{4a}{V}J_{2}^{*}\\ \frac{4a}{V}J_{2}^{*}\\ -\frac{4Td}{F}X_{\phi }^{*} \end{array}\right],\;\left[\begin{array}{c} J_{1}^{*}\\ J_{2}^{*}\\ J_{3}^{*}\\ X_{\phi }^{*} \end{array}\right]=\left[\begin{array}{cccc} \ell_{11}^{*} & \ell_{12}^{*} & \ell_{13}^{*} & \ell_{14}^{*}\\ \ell_{21}^{*} & \ell_{22}^{*} & \ell_{23}^{*} & \ell_{24}^{*}\\ \ell_{31}^{*} & \ell_{32}^{*} & \ell_{33}^{*} & \ell_{34}^{*}\\ \ell_{41}^{*} & \ell_{42}^{*} & \ell_{43}^{*} & 0 \end{array}\right]\left[\begin{array}{c} \frac{R}{d}\ln\left(\frac{c_{{\mathrm{Na}}^{+}}^{C}c_{{\mathrm{Cl}}^{-}}^{C}}{c_{{\mathrm{Na}}^{+}}^{D}c_{{\mathrm{Cl}}^{-}}^{D}}\right)\\ \frac{R}{d}\ln\left(\frac{c_{{\mathrm{Mg}}^{2+}}^{C}{\left(c_{{\mathrm{Cl}}^{-}}^{C}\right)}^{2}}{c_{{\mathrm{Mg}}^{2+}}^{D}{\left(c_{{\mathrm{Cl}}^{-}}^{D}\right)}^{2}}\right)\\ \frac{R}{d}\ln\left(\frac{c_{{\mathrm{SO}}_{4}^{2-}}^{C}{\left(c_{{\mathrm{Cl}}^{-}}^{D}\right)}^{2}}{c_{{\mathrm{SO}}_{4}^{2-}}^{D}{\left(c_{{\mathrm{Cl}}^{-}}^{C}\right)}^{2}}\right)\\ j/F \end{array}\right]. \end{array}$$

A linear least squares method was set up to regress the transport coefficients. Central to the method is the linearisation of the time derivative:

(30) $$\partial_{t}c_{i}^{D}\approx \frac{\Delta c_{i}^{D}}{\Delta t}$$

such that all measured concentrations, $c_{i}^{D}$, can be related to the fluxes and the time steps. In Appendix D it is shown how the ion concentrations and the OCV measurements can be collected in a vector of measurement data, *y*, and how the electric current and chemical potential gradients, through the transport equations, can be combined to a matrix *A*. This gives a matrix equation which reads

(31) $$\begin{array}{c} y=A\beta +\varepsilon , \end{array}$$

where β is a vector that contains the regression coefficients and ε is the error in the regression model. We note that the $\ell^{*}$-matrix can be described as linear functions of the regression parameters β when the electric conductance is omitted from $\ell^{*,\mathrm{ideal}}$ in Equation (24). In the case studies, we also explored regression without the constraints of the ideal ion selectivity assumption. In this model, we make no assumptions about the elements in $\ell^{*}$; we simply relax the constraints, except for keeping the Onsager symmetry. A few tests with these relaxed constraints showed that the model, with 9 free parameters, appeared to overfit the data. Thus, one element, $\ell_{23}^{*}$, which was seen to be small and to have a large covariance, was pruned. This leaves 8 parameters for the regression in the relaxed model as described in Equation (32), twice as many as for the ideal ion-selective model described in Equation (33).

(32) $$\begin{array}{c} \ell^{*,\mathrm{relaxed}}=\left[\begin{array}{cccc} P_{1} & P_{2} & P_{4} & P_{6}\\ P_{2} & P_{3} & 0 & P_{7}\\ P_{4} & 0 & P_{5} & P_{8}\\ P_{6} & P_{7} & P_{8} & N/A \end{array}\right] \end{array}$$

(33) $$\begin{array}{c} \ell^{*,\mathrm{ideal}}=\left[\begin{array}{cccc} 4P_{1} & -2P_{1} & 0 & P_{3}\\ -2P_{1} & P_{1} & 0 & \frac{1}{2}\left(1-P_{3}\right)\\ 0 & 0 & P_{2} & P_{4}\\ -P_{3} & -\frac{1}{2}\left(1-P_{3}\right) & -P_{4} & N/A \end{array}\right] \end{array}$$

The way the parameters appear in the $\ell^{*}$-matrix defines what we refer to as the ideal ion-selective model and the relaxed model. In the linear least squares method we then have $\beta^{\mathrm{ideal}}=\left[P_{1},P_{2},P_{3},P_{4}\right]$ and $\beta^{\mathrm{relaxed}}=\left[P_{1},P_{2},P_{3},P_{4},P_{5},P_{6},P_{7},P_{8}\right]$. Additionally, the initial ion concentrations for the relaxed model were set to be regression parameters. This was done since the measured concentration at time t=0 contained uncertainty, and we saw it as beneficial to give the model this degree of freedom. The implementation of this extra degree of freedom is described in Appendix D. The ideal ion-selective model was not given this degree of freedom.

The measurements [32] were taken at intervals which were quite long in comparison to what a numerical regression fit would benefit from. Plots were therefore generated from simulations using coefficients that were derived from fits according to Equation (29). The simulations were performed using a Runge-Kutta 4 method and much shorter time steps.

**Figure 2:**
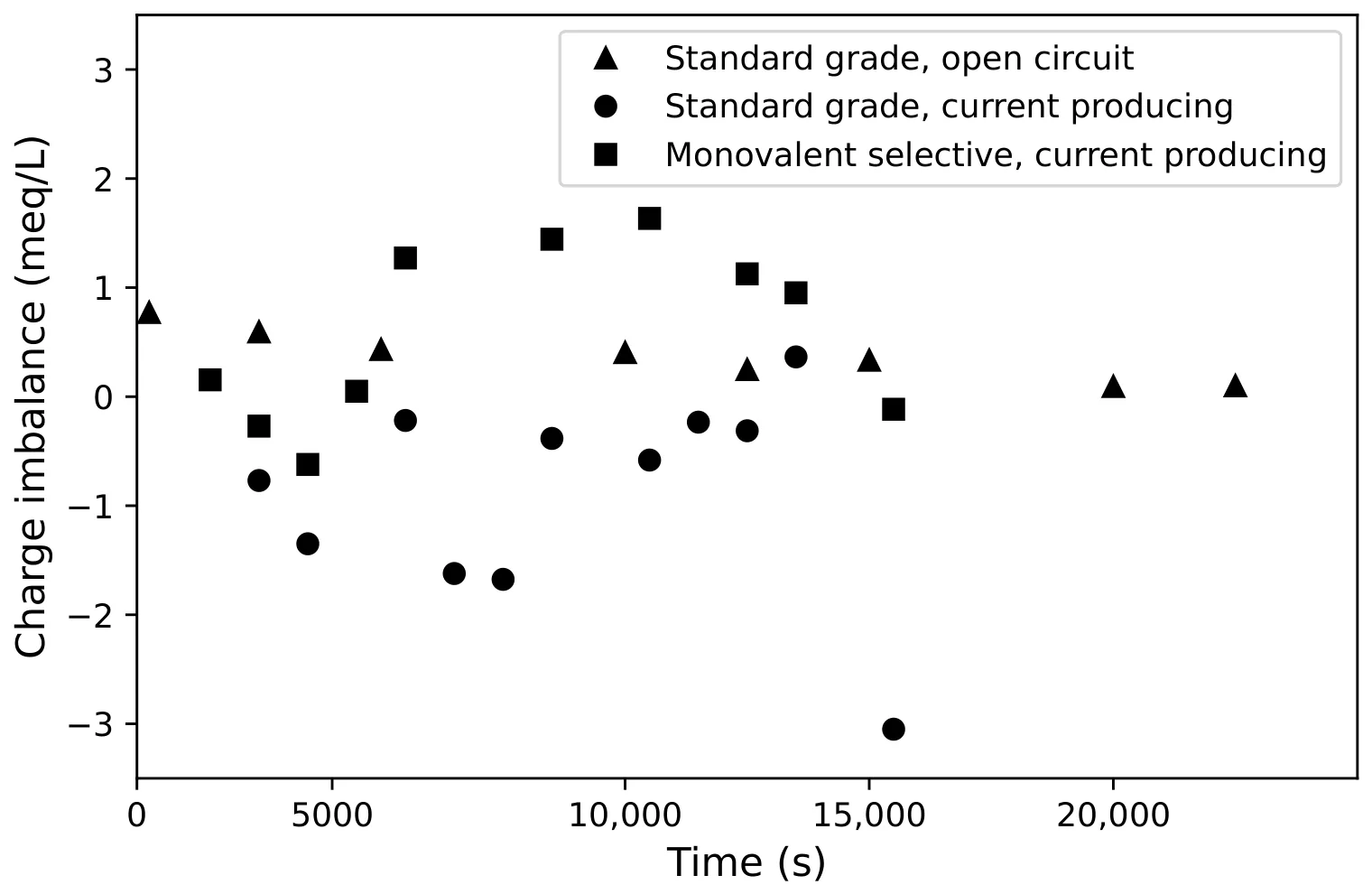
The charge imbalance, $\sum_{i}c_{i}z_{i}$, plotted for the three experimental measurement series used in this work.

The variances of the data points were not reported. Therefore, we used the charge balance to obtain some indication of the variance in the data sets. The electrolyte should follow the principle of electroneutrality (considering constant pH in the solutions), so the sum $\sum_{i}c_{i}z_{i}$ can be used to indicate the precision of the reported data set. For the measurement series used in this work, the deviation from electroneutrality is plotted in Figure 2. It shows that the charge imbalance is of the same order of magnitude as the concentration of divalent ions. We shall assume that the observed deviation from electroneutrality is mainly due to measurement variance for ${\mathrm{Na}}^{+}$ and ${\mathrm{Cl}}^{-}$, and therefore that the concentration trends for the divalent ions are statistically significant.

When experimental uncertainties are available, they are the natural choice for weights in the linear least squares regression. Here, since such uncertainties are lacking, we use the average of the measurement series as weights. We present the transport coefficients with the confidence interval of 2σ, which depends on the chosen weights. It is described in Appendix D how the variances, $\sigma^{2}$, are calculated in the weighted linear least squares method.

### 3.7. Case Studies

The aforementioned experimental data were fitted to effective transport coefficients. Four regressions were carried out. The first two considered both current-producing conditions and open-circuit conditions simultaneously. These first two regressions considered data from the experiments with the standard-grade RED stack, and the results are used to compare the ideal ion-selective model and the relaxed model. The last two regressions were performed with the ideal ion-selective model and only for current-producing conditions. One of the regressions considered the standard-grade RED stack, and the other regression considered the monovalent-selective RED stack. Results from the last two regressions are used to illustrate how the ideal ion-selective model can be used directly to estimate total ion mobilities and membrane resistance for both CEM and AEM, which can be compared for the two different RED stacks. The purpose of the case studies is to answer the questions, (I) “*Can the linear flux-force relations and their Onsager coefficients explain the uphill transport of MgSO_*4*_?*”, and (II) “*If so, how much can be explained by ideal ion selectivity behaviour and at which point is this approximation deemed too crude?*”.

## 4. Results

**Figure 3:**
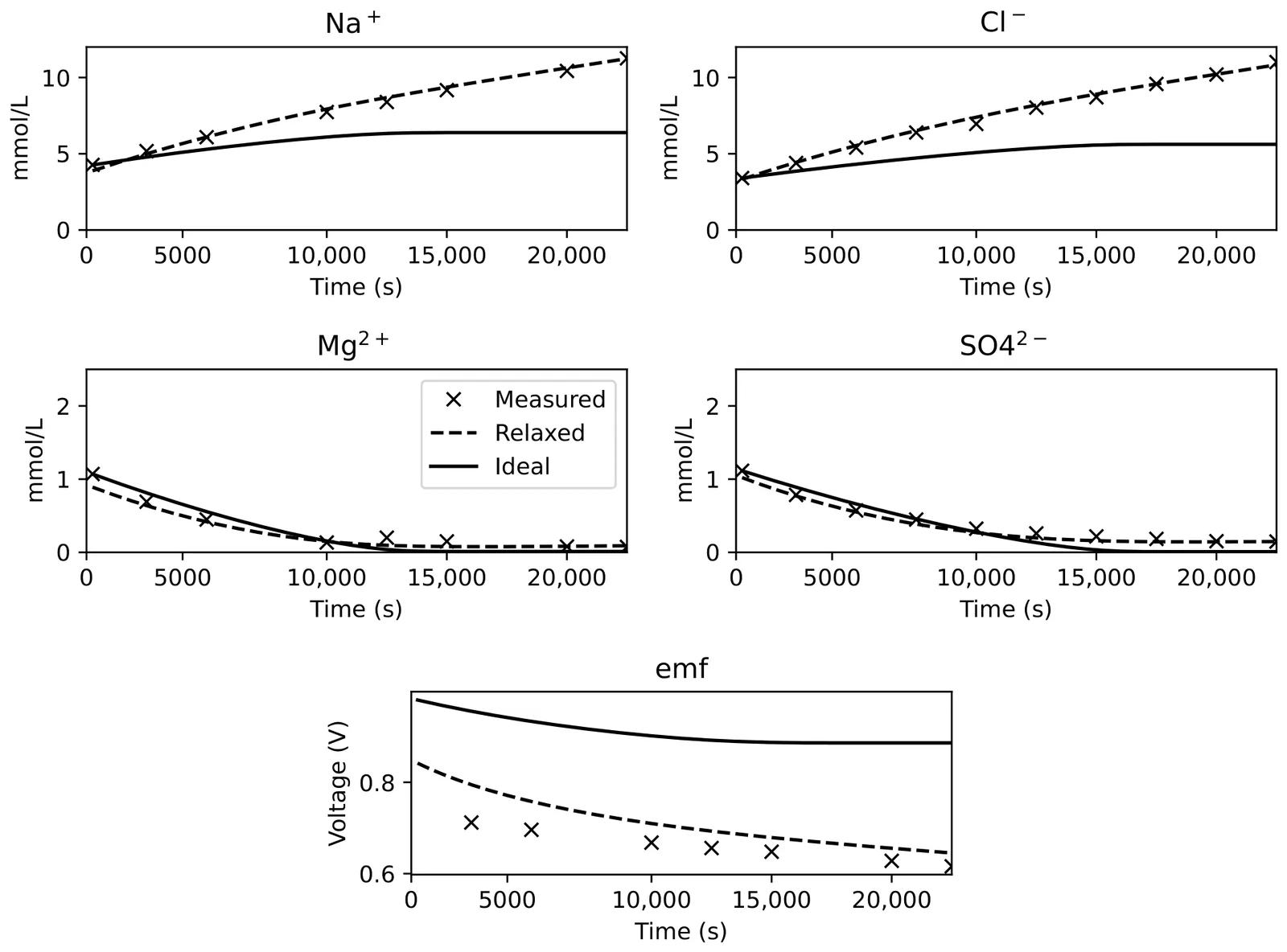
Fits of all experimental data (open-circuit and current-producing) of Post et al. [32] (crosses) to the relaxed model (stippled lines) and ideal ion selectivity model (whole line). The four top figures show the ion concentrations, and the bottom figure shows the emf variation. Results are from open-circuit conditions.

**Figure 4:**
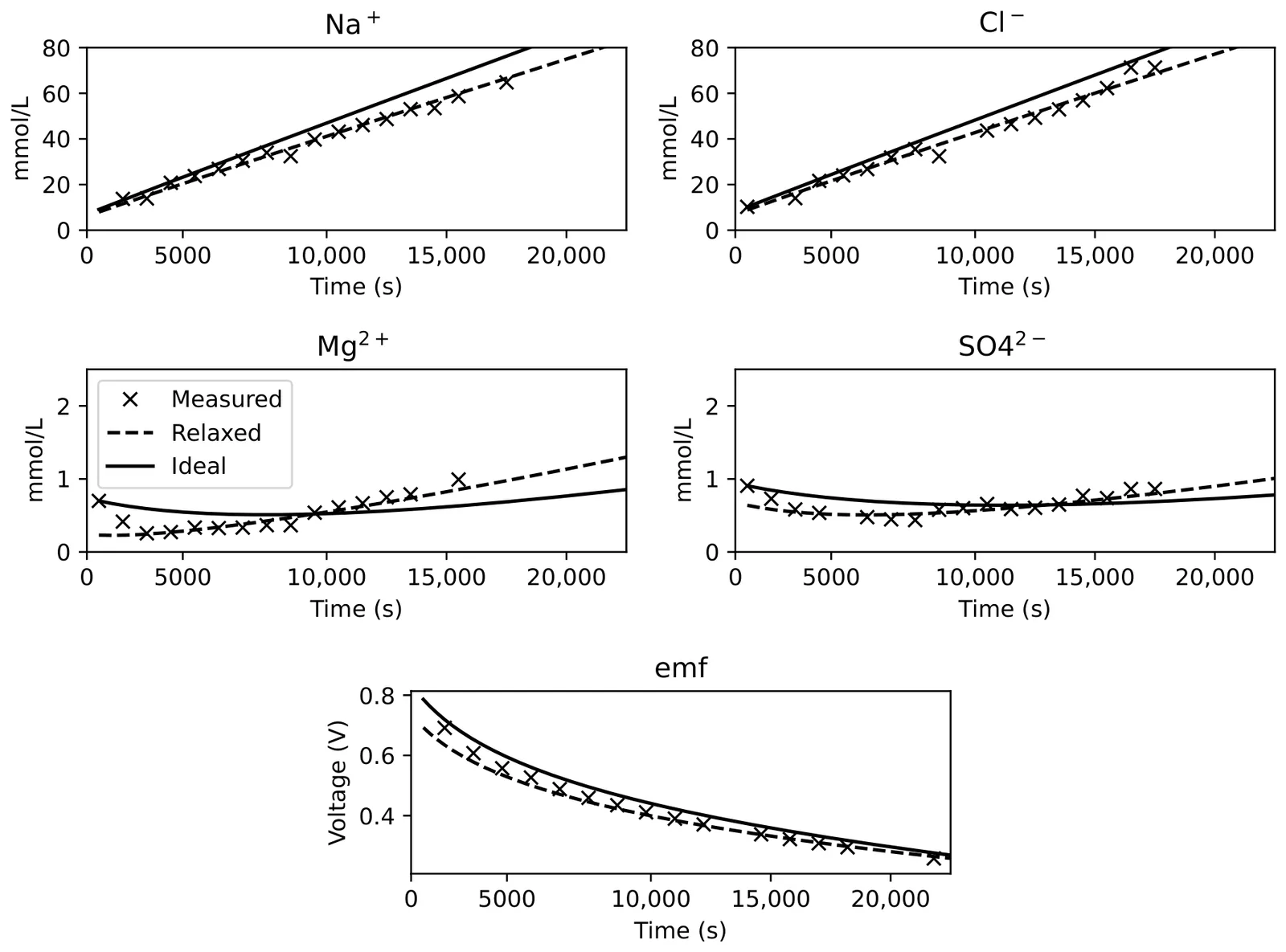
Fits of all experimental data (open-circuit and current-producing) of Post et al. [32] (crosses) to the relaxed model (stippled lines) and ideal ion selectivity model (whole line). The four top figures show the ion concentrations, and the bottom figure shows the emf variation. The results are from current-producing conditions.

Transport coefficients were fitted to align with the experimental data of the current-producing experiment and open-circuit experiments simultaneously for the standard-grade membrane. Results for the effective transport coefficients are presented in Equations (34) and (35) for the ideal ion selectivity and the relaxed model, respectively, with two times the estimated standard error of the parameter in the fitting. Simulations of concentration and voltage development were run with these transport coefficients, and the results are shown in Figure 3 and Figure 4, for open-circuit conditions and for current-producing conditions, respectively.

(34) $$\begin{array}{c} \ell^{*,\mathrm{ideal}}=\left[\begin{array}{cccc} \;\;\;42\times 10^{-12} & -21\times 10^{-12} & 0 & 1\\ -21\times 10^{-12} & \;\;\;10\times 10^{-12} & 0 & \;\;\;0.001\\ 0 & 0 & 8.3\times 10^{-12} & -0.002\\ -1 & -0.001 & 0.002 & 0 \end{array}\right],\;2\sigma =\left[\begin{array}{cccc} 3\times 10^{-12} & 2\times 10^{-12} & 0 & 0.01\\ 2\times 10^{-12} & 1\times 10^{-12} & 0 & 0.003\\ 0 & 0 & 0.8\times 10^{-12} & 0.002\\ 0.01 & 0.003 & 0.002 & 0 \end{array}\right] \end{array}$$

(35) $$\begin{array}{c} \ell^{*,\mathrm{relaxed}}=\left[\begin{array}{cccc} \;\;\;70\times 10^{-12} & -27\times 10^{-12} & \;\;4\times 10^{-12} & \;0.83\\ -27\times 10^{-12} & \;\;\;15\times 10^{-12} & 0 & \;\;\;0.008\\ \;\;4\times 10^{-12} & 0 & 16\times 10^{-12} & -0.003\\ -0.83 & -0.008 & 0.003 & 0 \end{array}\right],\;2\sigma =\left[\begin{array}{cccc} 10\times 10^{-12} & 6\times 10^{-12} & 1\times 10^{-12} & 0.03\\ \;\;6\times 10^{-12} & 3\times 10^{-12} & 0 & 0.003\\ \;\;1\times 10^{-12} & 0 & 4\times 10^{-12} & 0.003\\ 0.03 & 0.003 & 0.003 & 0 \end{array}\right] \end{array}$$

**Figure 5:**
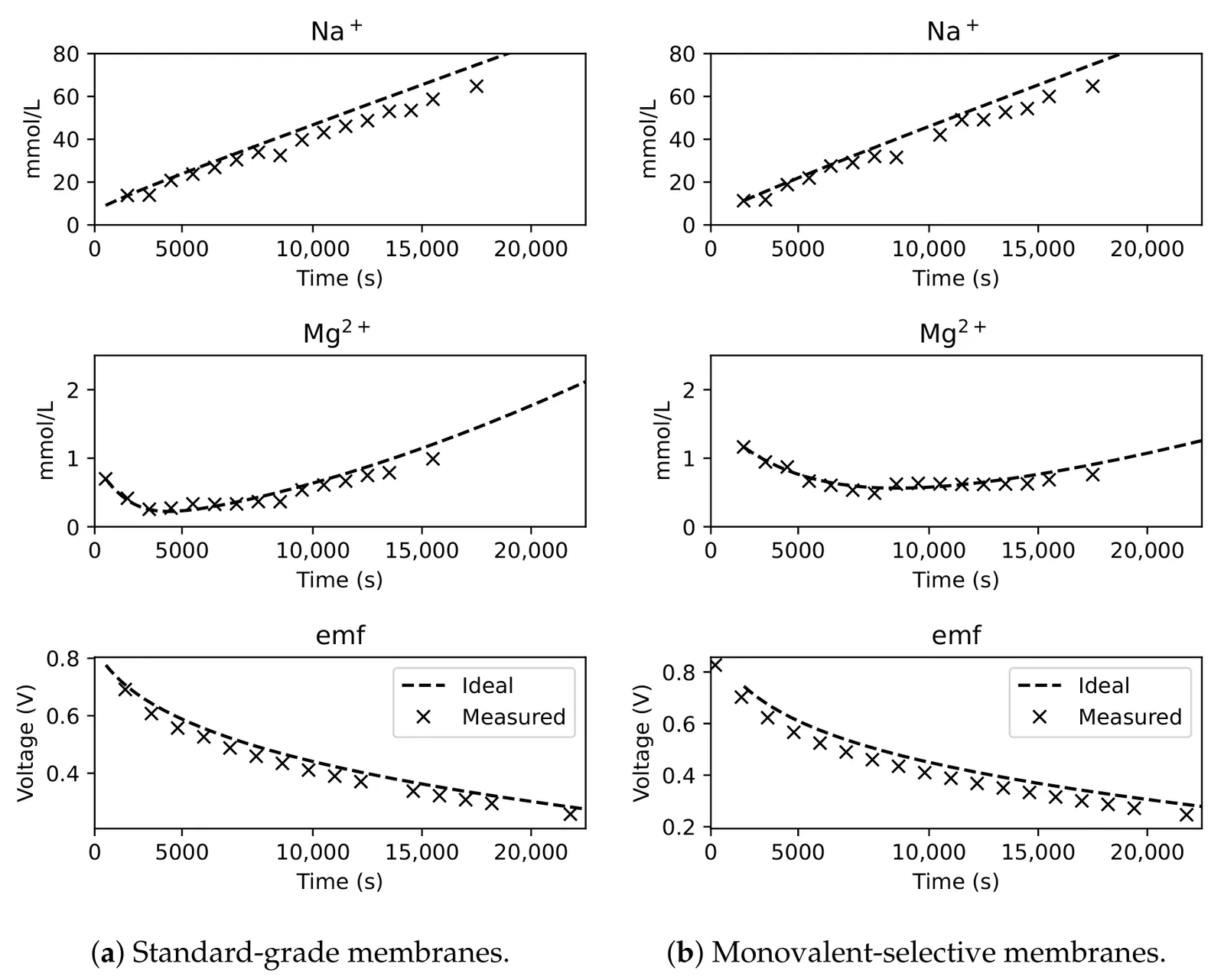
Simulation results showing cation fluxes and emf as functions of time fitted only to the current-producing condition measurements and with the ideal ion selectivity model, presented for ionic selective (**a**) and mono-selective (**b**) membranes. The top two figures show the concentration development of the cations, and the bottom figure shows the emf variation.

The ideal ion selectivity model was used to estimate the ion mobilities in the current-producing experiments with both the standard-grade membrane and the monovalent-selective membranes. The transport coefficients were then derived from the fits, this time only considering the current-producing experiment. The transport coefficients for the two membrane types are presented in Table 2, where *R* is the membrane resistance. The ion mobilities and membrane conductivities were derived from the fitted $\ell^{*}$-matrices. From the ion mobilities, the membrane conductivity, element $\ell_{44}^{*}$, was calculated according to the ideal ion selectivity equations. Although the model gave larger uncertainties for the monovalent-selective membranes (some confidence intervals include zero), both simulations gave good alignment with the measurements. The concentration development of sodium and magnesium, as well as the emf, is shown in Figure 5; figures including the anion concentrations are presented in Appendix C.

## 5. Discussion

### 5.1. A Model for Uphill Transport of Ions

This study set out to find an explanation for the uphill transport observed in the literature, in particular in the experiment of Post et al. [32]. From the results in Figure 3 and Figure 4, it is seen that our model is able to fit and describe the observed uphill transport of magnesium and sulfate. This follows from the observed reduction in concentration in the dilute solution compartment, in the presence of a positive concentration gradient in the direction of transport. The events are captured by the $\ell^{*}$-matrix of the relaxed model, as well as by the model of ideal ion selectivity. In the ideal ion selectivity model, there are only four parameters, all described by single ion mobilities. Uphill transport should not be considered a surprise from the NET theory. Rather, it is a phenomenon predictable from this theory from flux dependencies. Førland et al. [46] and Ratkje [47] gave similar coefficient relations for another ideal case. The transport properties follow from a systematic theory.

Uphill transport takes place as the fastest way for the system to decrease its overall Gibbs energy. The exchange of ${\mathrm{Mg}}^{2+}$ for ${\mathrm{Na}}^{+}$ in the dilute solution compartment occurs through a CEM (assuming negligible anion transport) as long as $\Delta \mu_{\mathrm{Mg}{\mathrm{Cl}}_{2}}-2\Delta \mu_{\mathrm{NaCl}}<0$. The phenomenon can be reversed by changing the salt concentrations [40]. This ion exchange is much quicker than co-ion leakage, and although $\Delta \mu_{\mathrm{Mg}{\mathrm{Cl}}_{2}}$ increases during the process, $\Delta \mu_{\mathrm{NaCl}}$ decreases faster. If there is co-ion leakage of ${\mathrm{Cl}}^{-}$, but not ${\mathrm{SO}}_{4}^{2-}$ through the CEM, the predictor for uphill transport of ${\mathrm{Mg}}^{2+}$ is instead $\ell_{22}^{*}\Delta \mu_{\mathrm{Mg}{\mathrm{Cl}}_{2}}-\ell_{21}^{*}\Delta \mu_{\mathrm{NaCl}}<0$. This illustrates that both the chemical potential differences and the magnitude of the diffusion coefficients are important for the direction and magnitude of uphill transport. This phenomenon is indeed similar to Donnan dialysis, as pointed out by Post et al. [32], and also shown experimentally by Emdadi et al. [40].

### 5.2. The Ideal Ion Selectivity vs. The Relaxed Model

The results have clearly shown that the ideal ion selectivity model predicts uphill transport, cf. Figure 3. The model has shortcomings, however, when it comes to describing experiments performed at open-circuit conditions. After roughly 10,000 s, simulations with the ideal ion selectivity model show that the sodium and chloride ion concentrations flatten out, whereas experimental results show a continued increase at this point. The plateauing is a trademark of the ideal ion selectivity model, as the model implies that $J_{{\mathrm{Na}}^{+}}+2J_{{\mathrm{Mg}}^{2+}}=0$ and $J_{{\mathrm{Cl}}^{-}}+2J_{{\mathrm{SO}}_{4}^{2-}}=0$ under open-circuit conditions. Co-ions are then not allowed to leak through the membrane. Thus, the observed continued increase of sodium and chloride ions is evidence of membrane non-ideality in terms of co-ion leakage (anions in CEM and cations in AEM) or water transport.

In Figure 3, we see a clear discrepancy between the measured data and the results from the ideal ion selectivity model for the emf. Recall that in the ideal ion selectivity model, the effective transport coefficients are restricted to $\ell_{14}^{*}+\frac{1}{2}\ell_{24}^{*}=1$. When this constraint is removed in the relaxed model, the emf aligns far better with experimental results.

The current-producing experiments show better agreement with the ideal ion selectivity model than experiments without net current. Circumstances are such that the ideal behaviour dominates the picture when j≠0; the contribution from diffusion becomes relatively smaller. It is seen that the ideal ion selectivity model describes measurements well, for standard-grade as well as monovalent-selective membranes in Figure 5.

The numerical values of the transport coefficients, shown in Equations (34) and (35), are interesting. Even when the strict constraints of the ideal ion selectivity model are lifted, and more coupling coefficients are introduced, the resulting relaxed model has the structure of the ideal $\ell^{*}$-matrix. Characteristic is that the matrix element $\ell_{13}^{*,\mathrm{relaxed}}$ is small compared to the other elements, and that $\ell_{11}^{*,\mathrm{relaxed}}\approx 4\ell_{22}^{*,\mathrm{relaxed}}$ and $\ell_{12}^{*,\mathrm{relaxed}}\approx -2\ell_{22}^{*,\mathrm{relaxed}}$. This is what we mean when we speak of the model pertaining to a structure in the coefficients. It is an interesting point that can be pursued with experiments on other membranes. The numerical values of the coefficients are of the same magnitude for both models, but the difference between diffusion coefficients in the relaxed model is between 1.5 and 1.7 times larger than the ones in the ideal model.

One advantage of the ideal ion selectivity model is that it allows us to extract CEM and AEM ion mobilities. These total ion mobilities contain the particular membrane ion concentration, cf. Equation (21). They are membrane-specific coefficients, but depend on the external salt solution.

### 5.3. Monovalent vs. Standard-Grade Membrane Values

It can be seen from Figure 5 that the ideal ion selectivity model captures the expected different behaviour of the investigated membrane types: the standard-grade membrane and the monovalent-selective membrane. The computed mobilities, transport numbers and resistances for the two membrane types are presented in Table 2. Comparing the ${\mathrm{Na}}^{+}$ and ${\mathrm{Mg}}^{2+}$ mobilities in this table, we see that the ideal model captures the larger monovalent selectivity of Na^+^ in the monovalent-selective membrane. In this membrane, there is a marked increase in the resistances of the results. The variance in the estimated values for the monovalent-selective membranes is large, however. This can be caused by inaccurate measurements or by inaccuracies in the model, which will be discussed further below.

Parameters like the ones presented in Table 2 demonstrate potential applications of the results. The RED process can be modelled in detail using the equations we have derived in combination with the transport properties obtained.

### 5.4. Applications to RED Stack Theory and Experiment

The ideal or the relaxed model, in combination with appropriately chosen transport coefficients, can be used to simulate the various real RED stack processes. If new single membrane mobilities could be made available, they could be readily introduced in the transport equations and used to compute RED stack performance under different operating conditions. For instance, we can obtain information on concentration variations over time, but also dissipation of energy in terms of local and global values of entropy production. Effects of new materials can thus be tested.

In this work, we have considered experiments performed on a RED stack. Single membrane mobilities can be obtained with higher accuracy using one membrane at a time. Such experiments could additionally allow determinations of anion mobilities through CEM and cation mobilities through AEM. With a few simple adjustments, the model presented can also be used directly and predict more precisely the RED stack behaviour. The overall theoretical and experimental setup then provides an accurate and reliable pathway to estimate power output and power dissipation of a RED stack.

### 5.5. Model Improvements

The theory chosen for the present transport process, non-equilibrium thermodynamics (NET), has proven its validity and value over many years and in numerous contexts. The foundations were spelt out in the last century by Onsager in his famous work from 1931. We are building on the adaptation to membrane transport by Katchalsky and Curran [3] and Førland et al. [46]; see also [45] for relevant and newer references. We have not added new theoretical elements to these texts, but we have taken advantage of an element which has not been put to use before in the present context. This element is the concept of entropy production invariance. It has been used earlier to relate one set of diffusion coefficients (Fick’s) to another set, the Maxwell–Stefan coefficients [48]. Here, it is used to relate sets of fluxes that describe transport in ion exchange membranes.

We have thus suggested a new example of an application of this concept by linking the Nernst–Planck equations in their simplest form to a form which allows for coupling of fluxes in agreement with Onsager. The choice of neutral salts as components to describe the second system brings the NET theory close to the experimental situation. This advantage was first recognised and pioneered by Kedem and Katchalsky [3]. It was later advocated by Førland et al. [46]. The advantage of having a theory directly relatable to experiments is invaluable for the decomposition of experimental data. Finally, the possibility of control of assumptions made in theory is important. Some additional assumptions will be discussed below. These can be relaxed in the future.

#### 5.5.1. Activity Coefficient Considerations

The effect of water transport and activity coefficients can be addressed by estimating their influence on the calculated emf. The main term in the emf, from the chemical potential difference of sodium chloride, is proportional to $\ln\left(\frac{c_{{\mathrm{Na}}^{+}}^{C}c_{{\mathrm{Cl}}^{-}}^{C}}{c_{{\mathrm{Na}}^{+}}^{D}c_{{\mathrm{Cl}}^{-}}^{D}}\right)+\ln\left(\frac{\gamma_{{\mathrm{Na}}^{+}}^{C}\gamma_{{\mathrm{Cl}}^{-}}^{C}}{\gamma_{{\mathrm{Na}}^{+}}^{D}\gamma_{{\mathrm{Cl}}^{-}}^{D}}\right)$. The contribution from the activity coefficients was disregarded (assuming it was constant) in this study, and is thus a source of error. For the given initial concentrations of NaCl, the mean molal dilute and concentrated activity coefficients are around 0.8 and 0.7 [49]. Comparing the logarithmic term from activity coefficient differences with the logarithmic term from concentration differences, the activity coefficient term is about 4% of the concentration term. The inclusion of activity coefficients could thus be expected to cause about a 4% decrease in the calculated emf.

#### 5.5.2. Water Flux Considerations

When water is transferred between two solutions, both ion concentrations and the volume subsequently change. An argument against the present theory could be that if magnesium is not transferred and water displaces, one will see an apparent uphill transport of magnesium. In other words, if magnesium is immobile and water moves into the dilute compartment, it will appear as if magnesium is transported uphill. This is, however, not the case, as discussed below.

In ED and RED, water can be transferred by two mechanisms: osmosis (diffusion of water from dilute to concentrated) and electro-osmosis (water transfer by coupling to ion transport via water transference coefficient $t_{w}$, from concentrated to dilute). Considering electro-osmosis, we found in Section 2 that the volume changes in the reactive solutions are up to 2%. For pure osmosis, we have also assessed this to be up to a 9% volume change for these solutions. These contributions are in opposite directions such that the worst case estimate is still 9%. This increase (decrease) in concentrate (dilute) volume would affect the salt concentration and the first ln-term above could be decreased by up to 8%. Note here that 8% would be the estimate for the end of the experiment. At the beginning of the experiment, no water has yet been transported. From all case studies, Figure 3, Figure 4 and Figure 5, it is seen that the calculated emf from the ideal ion selectivity model is higher than the measured values. Inclusion of activity coefficients and water transport is thus identified as a possible improvement to the model.

Membrane-solution equilibria and accumulation of ions and water inside the membrane have also not been included in the current model. Ion exchange membranes tend to preferentially bind divalent ions over monovalent ones [34,35], which may explain some of the (early) disappearance of divalent ions from the dilute compartment. It is, however, more likely that the membrane accumulates divalent ions from the concentrate compartment. The measurement error seen in the deviation from local electroneutrality in the dilute compartment, Figure 2, is likely a larger source of error in the parameter estimation. Some aspects that may aid future studies on membrane transport phenomena are simultaneous measurements of volume, more frequent measurements at the start when uphill transport occurs, and single membrane diffusion experiments. Also, single membrane experiments should be done. Overall, the exact values from the parameter estimation carry uncertainty due to measurement errors and model simplifications. The parameter trends and the explanatory ability of even the ideal ion selectivity model are still useful.

In order to deal with water in a quantitative manner, one has to include a flux-force term in the entropy production of the system. This will introduce one more flux equation to solve, with more cross-coefficients to include. The practical influence of the water flux is a volume change over time, which would have to be measured. This was not measured by Post et al. and is therefore difficult to include in this analysis. We have wanted first to document how far it is possible to come with an ideal model and one way to relax it.

## 6. Conclusions

Reflecting on the results and discussion sections, we conclude the following:

- Non-equilibrium thermodynamic theory (NET) is able to capture uphill transport of magnesium ions between solutions with different compositions of the salts NaCl and MgSO_4_. Such transport may take place in sea–river water RED stack processes.
- The NET procedure was applied to two models: a simple model with ideal ion selectivity and four transport coefficients, and a relaxed model with eight transport coefficients. The ideal model assumes no co-ion leakage in the membranes. The relaxed model allows for such leakage and therefore explains diffusion experiments better. The model explains diffusion and charge transfer as superimposed. Diffusion is relatively insignificant compared to migration in the simulations of experiments with net electric current.
- A new method of analysis has been demonstrated for electrolyte solutions, taking advantage of entropy production invariance. This method could be used to help build data banks for IEMs. It may also improve existing modelling of RED stack performance in addition to helping design new experiments.

The invariance of the entropy production has been used for the first time in order to relate descriptions of transport in electrolyte solutions. The fit of experimental data to the models in question is therefore theoretically sound. A range of membrane transport coefficients is obtained for diffusion and charge transfer. The accuracy of the coefficient fit is hampered by the experimental uncertainty present in the measurements and simplifying assumptions, but the coefficient values are of the expected order of magnitude and allow for an explanation of uphill transport. The method presented here might well turn out to be a useful tool for the modelling of cell power and power losses, not only in RED, but in electrochemical systems at large. 

## Figures and Tables

**Table 1:** Relevant properties of the Neosepta membranes based on the manufacturer’s specification (Astom Co., formerly Tokuyama Co.), where area resistance is measured in a 0.5 mol L^−1^ NaCl solution at 25 °C [41]. Ion exchange capacities were reported by Wiśniewski et al. [42] and Rottiers et al. [43].

	Standard Grade			Monovalent Selective	
	**Neosepta CMX**	**Neosepta AMX**		**Neosepta CMS**	**Neosepta ACS**
Main functional group	-${\mathrm{SO}}_{3}^{-}$	-N^+^R_3_		-${\mathrm{SO}}_{3}^{-}$	-N^+^R_3_
Thickness, *d* (mm)	0.17	0.14		0.15	0.13
Ion exchange capacity (meq ·g^−1^)	1.5–1.8 [42]	1.4–1.7 [42]		2.0–2.5 [43]	1.4–2.0 [42]
Reference resistance (Ωcm^2^)	3	2.4		1.8	3.8

**Table 2:** Estimated transport properties for the standard-grade membrane and for the monovalent-selective membrane found from the current-producing experiment.

	Standard Grade	Monovalent Selective
$\tau_{{\mathrm{Na}}^{+}}$	0.96 ± 0.01	1.00 ± 0.01
$\tau_{{\mathrm{SO}}_{4}^{2-}}$	0.007 ± 0.003	0.02 ± 0.01
$U_{{\mathrm{Na}}^{+}}F^{2}\left(\Omega^{-1}\right)$	60 ± 10	200 ± 300
$U_{{\mathrm{Mg}}^{2+}}F^{2}\left(\Omega^{-1}\right)$	0.7 ± 0.1	0.29 ± 0.06
$U_{{\mathrm{Cl}}^{-}}F^{2}\left(\Omega^{-1}\right)$	80 ± 30	20 ± 30
$U_{{\mathrm{SO}}_{4}^{2-}}F^{2}\left(\Omega^{-1}\right)$	0.28 ± 0.05	0.2 ± 0.2
$R^{\mathrm{CEM}}\left(\Omega {\mathrm{cm}}^{2}\right)$	1.5 ± 0.3	0.4 ± 0.6
$R^{\mathrm{AEM}}\left(\Omega {\mathrm{cm}}^{2}\right)$	1.2 ± 0.5	4 ± 5

**Figure A1:**
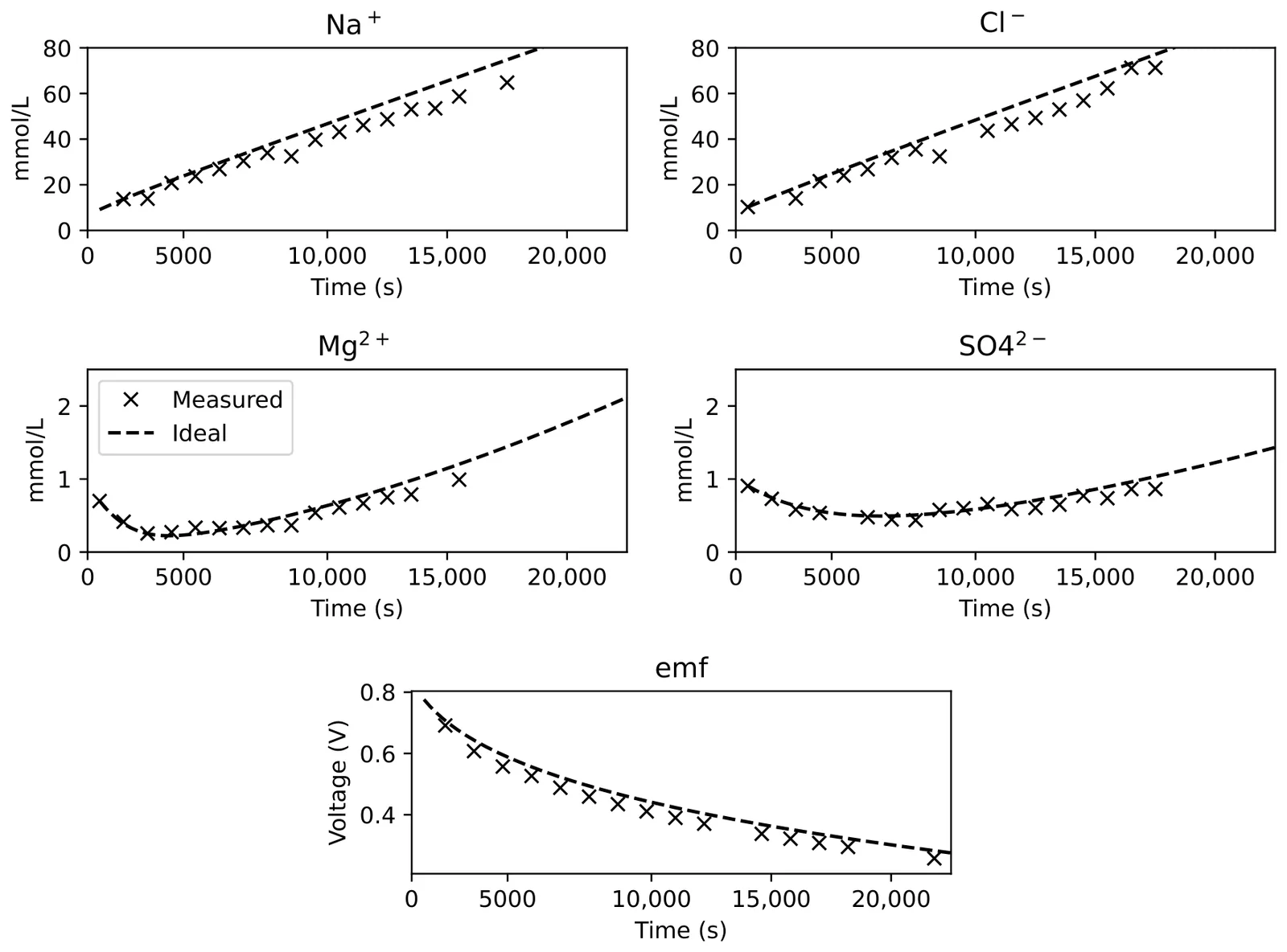
The simulation results of the standard-grade current-producing experiments. The four top figures show the ion concentrations and the bottom figure shows the emf variation.

**Figure A2:**
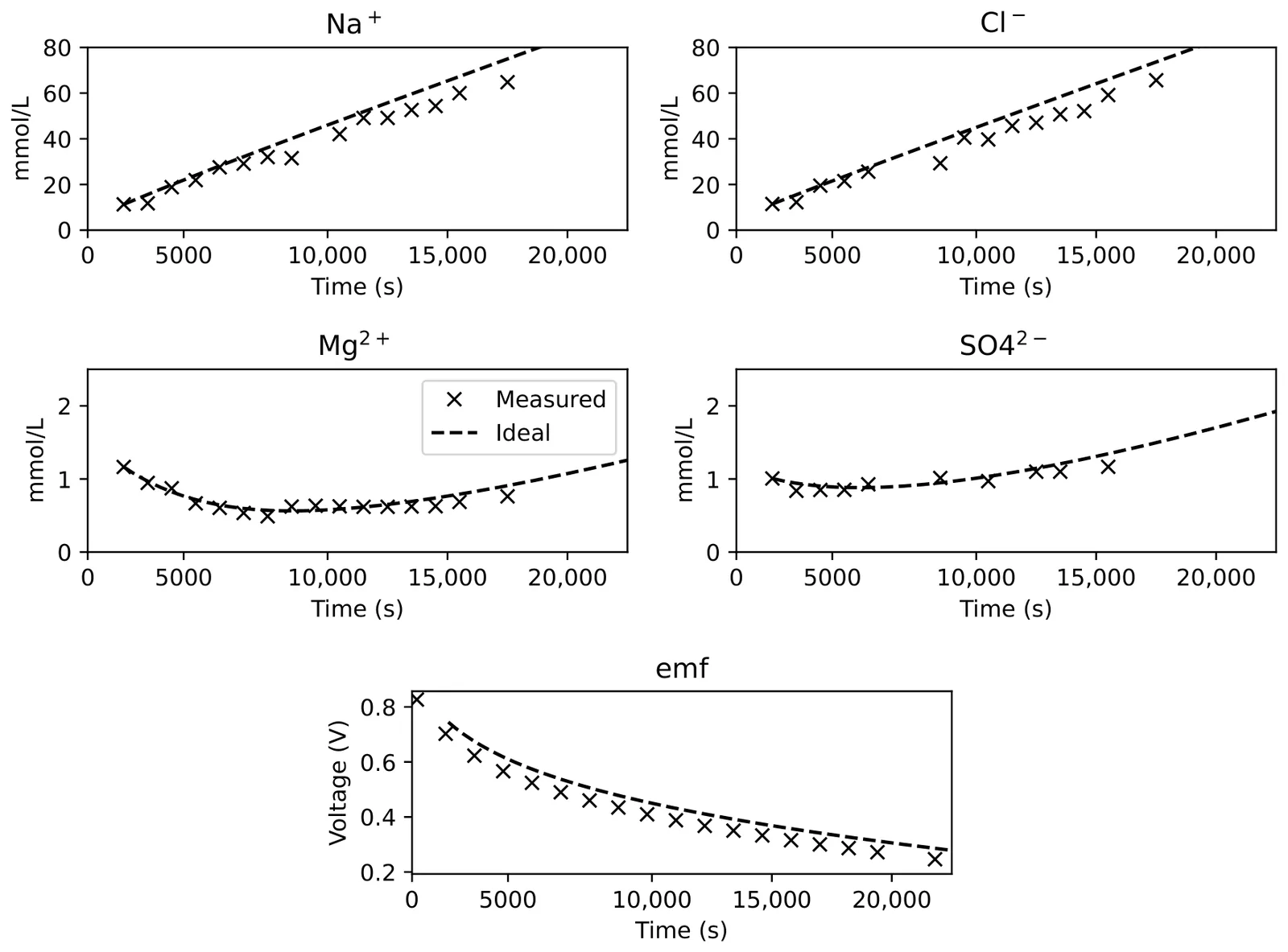
The simulation results of the monovalent-selective current-producing experiments. The four top figures show the ion concentrations, and the bottom figure shows the emf variation.

## Data Availability

The original data and figures are available from the corresponding author upon request.

## Appendix A. Osmosis and Electro-Osmosis Considerations

In the numerical study, the water transport was neglected. Water transport through the membranes would lead to a change in volume. Water transport can be divided into two terms. The first contribution comes from osmosis, where water is transported from high osmotic pressure to low osmotic pressure. The second contribution is electro-osmosis, meaning that the ions that are transported through the membrane are accompanied by water molecules. The regular osmosis would drive water from the dilute solution to the concentrate solution. The electro-osmosis, on the other hand, would cause water to move from the concentrated to the dilute solution alongside the ions. The latter would therefore mean an increased volume in the dilute solution, which would effectively cause a drop in magnesium and sulfate concentrations in the current-producing case. In the experiments on which we base these case studies, no measurements of volume were made, so potential modelled volume changes could not be validated in these cases. We shall, however, provide estimates of the inaccuracies that these effects can cause.

Firstly, consider electro-osmosis. By looking at the experimental data for the current-producing experiment, we can roughly estimate that the sodium and chloride concentrations change by 70 mmol/L during the experiment, whereas the change in magnesium and sulfate is negligible in comparison. As an estimate, we assume that every sodium and chloride ion carries 7 water molecules [50]. Using a molar volume of water of $v_{w}=18\cdot 10^{-3}$ L/mol, we can estimate the volume change from electro-osmosis, $\Delta V^{\mathrm{elec}}$, to be

(A1) $$\begin{array}{c} \Delta V^{\mathrm{elec}}=7\left(\Delta c_{\mathrm{Na}}^{D}+\Delta c_{\mathrm{Cl}}^{D}\right)v_{w}V=7\left(70\;\mathrm{mmol}/L+70\;\mathrm{mmol}/L\right)\cdot 18\cdot 10^{-3}\;L/\mathrm{mol}V\approx 0.02V. \end{array}$$

In this case, electro-osmosis contributes to a volume increase of roughly 2% in the dilute solution.

For diffusion osmosis, we use estimates of water permeability from the work of Moreno et al. [11]. This water permeability is reported for another type of ion exchange membrane. However, as no values were reported for the membranes considered in this work, the largest value reported by Moreno et al. is used in the estimates of the osmosis effect. The water permeability $L_{p}=15$ m L bar^−1^ h^−1^m^−2^ is used. The osmotic pressure is simplified as $P_{0}=cRT$, where *c* should be seen as the total ion concentration. With no hydrostatic pressure difference, the water flux can be modelled as $J_{w}=L_{p}RT\Delta c$, where Δc is the total ion concentration difference between concentrate and dilute solution. The total ion concentration difference is roughly 1 mol/L at the beginning of the experiment, and we use this as a worst-case estimate although it does change during the experiment. Using 22,500 s or 6.25 h as the length of the experiment, Δt=6.25 h, we estimate the volume change due to diffusion:

(A2) $$\begin{array}{cc} \Delta V^{\mathrm{diff}} & \approx 4aL_{p}RT\Delta c\Delta t\\ & =15\cdot 10^{-3}\;L{\mathrm{bar}}^{-1}h^{-1}m^{-2}\cdot 0.08314\;L\mathrm{bar}K^{-1}{\mathrm{mol}}^{-1}\cdot 298.15\;K\cdot 1\mathrm{mol}L^{-1}\cdot 4\cdot 0.01\;m^{2}\cdot 6.25\;h\\ & =0.09\;L. \end{array}$$

Here, *a* is the membrane area and is multiplied by 4 to account for all unit cells. As the solution volume is 1.05 L, this would mean a volume decrease of the dilute solution of about 9%.

## Appendix B. Transformations Between Ion and Neutral Basis

Here we formalise how to transform the transport coefficient matrix from the ion representation to our chosen representation, which was useful when applying the ideal ion selectivity assumption.

The first step in formalising this transformation is to formulate the transformation of fluxes and forces by transformation matrices

(A3) $$\begin{array}{cc} \; & J=T^{J}J^{\mathrm{ion}}, \end{array}$$

(A4) $$\begin{array}{cc} \; & X=T^{X}X^{\mathrm{ion}}, \end{array}$$

where

(A5) $$\begin{array}{c} T^{J}=\left[\begin{array}{cccc} 1 & 0 & 0 & 0\\ 0 & 0 & 1 & 0\\ 0 & 0 & 0 & 1\\ 1 & -1 & 2 & -2 \end{array}\right],\;\;\;\;\;\;\;\;T^{X}=\left[\begin{array}{cccc} 1 & 1 & 0 & 0\\ 0 & 2 & 1 & 0\\ 0 & -2 & 0 & 1\\ 0 & -1 & 0 & 0 \end{array}\right]. \end{array}$$

From this we can derive the transformation of the *L* matrices of the different representations

(A6) $$\begin{array}{cc} J^{\mathrm{ion}} & =L^{\mathrm{ion}}X^{\mathrm{ion}}, \end{array}$$

(A7) $$\begin{array}{cc} T^{J}J^{\mathrm{ion}} & =T^{J}L^{\mathrm{ion}}X^{\mathrm{ion}}, \end{array}$$

(A8) $$\begin{array}{cc} J & =T^{J}L^{\mathrm{ion}}{\left(T^{X}\right)}^{-1}X. \end{array}$$

In the second line we have multiplied by $T^{J}$ from the left, and on the third line we have used $X^{\mathrm{ion}}={\left(T^{X}\right)}^{-1}X$. From the third line, we can recognise

(A9) $$\begin{array}{c} L=T^{J}L^{\mathrm{ion}}{\left(T^{X}\right)}^{-1}. \end{array}$$

In order to transform $L^{\mathrm{ion}}$ to *L* we need the inverse of the $T^{X}$ matrix, which can be found to be

(A10) $${\left(T^{X}\right)}^{-1}=\left[\begin{array}{cccc} 1 & 0 & 0 & 1\\ 0 & 0 & 0 & -1\\ 0 & 1 & 0 & 2\\ 0 & 0 & 1 & -2 \end{array}\right].$$

## Appendix C. Simulations of Monovalent-Selective and Standard-Grade Experiments

Measurements and simulated results for emf and all ion concentrations are shown in Figure A1 for standard-grade membranes and Figure A2 for monovalent-selective membranes. These results were produced with the ideal ion selectivity model.

## Appendix D. Linear Fitting Scheme

This section explains the implementation of the linear least squares method used to estimate the transport coefficients. The first step is to linearise the time derivative and express the concentration of ion *i* in the *k*-th time step, $t_{k}$, by the concentration in the previous time step $t_{k-1}$ and the flux in time step *k* as follows:

(A11) $$\begin{array}{c} c_{i}\left(t_{k}\right)=c_{i}\left(t_{k-1}\right)+\frac{4a}{V}J_{i}\left(t_{k}\right)\Delta t_{k}, \end{array}$$

where *a* is the membrane area, V is the solution volume and $\Delta t_{k}=t_{k}-t_{k-1}$. Furthermore, the concentration at time $t_{k}$ is expressed by the initial concentration $c_{i}\left(t_{0}\right)$ and the ion flux at all times smaller than $t_{k}$:

(A12) $$\begin{array}{c} c_{i}\left(t_{k}\right)=c_{i}\left(t_{0}\right)+\sum_{m=1}^{k}\frac{4a}{V}J_{i}\left(t_{m}\right)\Delta t_{m}. \end{array}$$

This effectively sums up all of the concentration change due to a flux up to the measurement at time $t_{k}$. The vector of concentrations of an ion for all time steps can thus be written as:

(A13) $$\begin{array}{c} \left[\begin{array}{cccc} c_{i}\left(t_{1}\right) & c_{i}\left(t_{2}\right) & c_{i}\left(t_{3}\right) & \cdots \end{array}\right]=\left[\begin{array}{cccc} c_{i}\left(t_{0}\right) & c_{i}\left(t_{0}\right) & c_{i}\left(t_{0}\right) & \cdots \end{array}\right]+\frac{4a}{V}\left[\begin{array}{cccc} J_{i}\left(t_{1}\right) & J_{i}\left(t_{2}\right) & J_{i}\left(t_{3}\right) & \cdots \end{array}\right]\overset{\Delta t}{\overset{︷}{\left[\begin{array}{cccc} \Delta t_{1} & \Delta t_{1} & \Delta t_{1} & \cdots \\ 0 & \Delta t_{2} & \Delta t_{2} & \cdots \\ 0 & 0 & \Delta t_{3} & \cdots \\ \vdots & \vdots & \vdots & \ddots \end{array}\right]}}. \end{array}$$

which is merely all of the measurements of $c_{i}$ expressed in terms of Equation (A12) in matrix form. Repeating this operation for all of the measured ion concentrations and also the voltages at all the time steps yields:

(A14) $$\begin{array}{cc} \; & \left[\begin{array}{cccc} c_{{\mathrm{Na}}^{+}}\left(t_{1}\right) & c_{{\mathrm{Na}}^{+}}\left(t_{2}\right) & c_{{\mathrm{Na}}^{+}}\left(t_{3}\right) & \cdots \\ c_{{\mathrm{Mg}}^{2+}}\left(t_{1}\right) & c_{{\mathrm{Mg}}^{2+}}\left(t_{2}\right) & c_{{\mathrm{Mg}}^{2+}}\left(t_{3}\right) & \cdots \\ c_{{\mathrm{SO}}_{4}^{2-}}\left(t_{1}\right) & c_{{\mathrm{SO}}_{4}^{2-}}\left(t_{2}\right) & c_{{\mathrm{SO}}_{4}^{2-}}\left(t_{3}\right) & \cdots \\ c_{{\mathrm{Cl}}^{-}}\left(t_{1}\right) & c_{{\mathrm{Cl}}^{-}}\left(t_{2}\right) & c_{{\mathrm{Cl}}^{-}}\left(t_{3}\right) & \cdots \\ V(t_{1}) & V(t_{2}) & V(t_{3}) & \cdots \end{array}\right]=\overset{c^{0}}{\overset{︷}{\left[\begin{array}{c} c_{{\mathrm{Na}}^{+}}\left(t_{0}\right)\\ c_{{\mathrm{Mg}}^{2+}}\left(t_{0}\right)\\ c_{{\mathrm{SO}}_{4}^{2-}}\left(t_{0}\right)\\ c_{{\mathrm{Cl}}^{-}}\left(t_{0}\right)\\ 0 \end{array}\right]\left[\begin{array}{cccccc} 1 & 1 & 1 & 1 & 1 & \cdots \end{array}\right]}} \end{array}$$

(A15) $$\begin{array}{c} +\frac{4a}{V}\left[\begin{array}{cccc} J_{{\mathrm{Na}}^{+}}\left(t_{1}\right) & J_{{\mathrm{Na}}^{+}}\left(t_{2}\right) & J_{{\mathrm{Na}}^{+}}\left(t_{3}\right) & \cdots \\ J_{{\mathrm{Mg}}^{2+}}\left(t_{1}\right) & J_{{\mathrm{Mg}}^{2+}}\left(t_{2}\right) & J_{{\mathrm{Mg}}^{2+}}\left(t_{3}\right) & \cdots \\ J_{{\mathrm{SO}}_{4}^{2-}}\left(t_{1}\right) & J_{{\mathrm{SO}}_{4}^{2-}}\left(t_{2}\right) & J_{{\mathrm{SO}}_{4}^{2-}}\left(t_{3}\right) & \cdots \\ J_{{\mathrm{Cl}}^{-}}\left(t_{1}\right) & J_{{\mathrm{Cl}}^{-}}\left(t_{2}\right) & J_{{\mathrm{Cl}}^{-}}\left(t_{3}\right) & \cdots \\ 0 & 0 & 0 & \cdots \end{array}\right]\left[\begin{array}{cccc} \Delta t_{1} & \Delta t_{1} & \Delta t_{1} & \cdots \\ 0 & \Delta t_{2} & \Delta t_{2} & \cdots \\ 0 & 0 & \Delta t_{3} & \cdots \\ \vdots & \vdots & \vdots & \ddots \end{array}\right]-\frac{4dT}{F}\left[\begin{array}{cccc} 0 & 0 & 0 & \cdots \\ 0 & 0 & 0 & \cdots \\ 0 & 0 & 0 & \cdots \\ 0 & 0 & 0 & \cdots \\ X_{\phi }\left(t_{1}\right) & X_{\phi }\left(t_{2}\right) & X_{\phi }\left(t_{3}\right) & \cdots \end{array}\right]. \end{array}$$

Here, the concentrations at any time step depend directly on the time, but the voltage depends only indirectly on time and directly on the concentrations at any time. They are therefore split into two matrices on the right-hand side. Next, we express the ion fluxes with the transport coefficients and forces:

(A16) $$\begin{array}{cc} \frac{4a}{V}\; & \left[\begin{array}{cccc} J_{{\mathrm{Na}}^{+}}\left(t_{1}\right) & J_{{\mathrm{Na}}^{+}}\left(t_{2}\right) & J_{{\mathrm{Na}}^{+}}\left(t_{3}\right) & \ldots \\ J_{{\mathrm{Mg}}^{2+}}\left(t_{1}\right) & J_{{\mathrm{Mg}}^{2+}}\left(t_{2}\right) & J_{{\mathrm{Mg}}^{2+}}\left(t_{3}\right) & \ldots \\ J_{{\mathrm{SO}}_{4}^{2-}}\left(t_{1}\right) & J_{{\mathrm{SO}}_{4}^{2-}}\left(t_{2}\right) & J_{{\mathrm{SO}}_{4}^{2-}}\left(t_{3}\right) & \ldots \\ J_{{\mathrm{Cl}}^{-}}\left(t_{1}\right) & J_{{\mathrm{Cl}}^{-}}\left(t_{2}\right) & J_{{\mathrm{Cl}}^{-}}\left(t_{3}\right) & \ldots \\ 0 & 0 & 0 & \ldots \end{array}\right]=\overset{B^{c}}{\overset{︷}{\frac{4a}{V}\left[\begin{array}{cccc} 1 & 0 & 0 & 0\\ 0 & 1 & 0 & 0\\ 0 & 0 & 1 & 0\\ 1 & 2 & 2 & 0\\ 0 & 0 & 0 & 0 \end{array}\right]}}\overset{\ell^{*}}{\overset{︷}{\left[\begin{array}{cccc} \ell_{11}^{*} & \ell_{12}^{*} & \ell_{13}^{*} & \ell_{14}^{*}\\ \ell_{21}^{*} & \ell_{22}^{*} & \ell_{23}^{*} & \ell_{24}^{*}\\ \ell_{31}^{*} & \ell_{32}^{*} & \ell_{33}^{*} & \ell_{34}^{*}\\ \ell_{41}^{*} & \ell_{42}^{*} & \ell_{43}^{*} & 0 \end{array}\right]}}\overset{X}{\overset{︷}{\left[\begin{array}{cccc} X_{1}\left(t_{1}\right) & X_{1}\left(t_{2}\right) & X_{1}\left(t_{3}\right) & \ldots \\ X_{2}\left(t_{1}\right) & X_{2}\left(t_{2}\right) & X_{2}\left(t_{3}\right) & \ldots \\ X_{3}\left(t_{1}\right) & X_{3}\left(t_{2}\right) & X_{3}\left(t_{3}\right) & \ldots \\ j/F & j/F & j/F & \ldots \end{array}\right]}}. \end{array}$$

Note that the 4th row of the $\ell^{*}$ matrix does not affect the matrix on the left-hand side here, but it is included without the $\ell_{44}^{*}$ element, as we want to use the same matrix in the next part when we express the emf with the same matrix.

(A17) $$\begin{array}{cc} \; & -\frac{4dT}{F}\left[\begin{array}{cccc} 0 & 0 & 0 & \cdots \\ 0 & 0 & 0 & \cdots \\ 0 & 0 & 0 & \cdots \\ 0 & 0 & 0 & \cdots \\ X_{\phi }\left(t_{1}\right) & X_{\phi }\left(t_{2}\right) & X_{\phi }\left(t_{3}\right) & \cdots \end{array}\right]=\overset{B^{V}}{\overset{︷}{-\frac{4dT}{F}\left[\begin{array}{cccc} 0 & 0 & 0 & 0\\ 0 & 0 & 0 & 0\\ 0 & 0 & 0 & 0\\ 0 & 0 & 0 & 0\\ 0 & 0 & 0 & 1 \end{array}\right]}}\overset{\ell^{*}}{\overset{︷}{\left[\begin{array}{cccc} \ell_{11}^{*} & \ell_{12}^{*} & \ell_{13}^{*} & \ell_{14}^{*}\\ \ell_{21}^{*} & \ell_{22}^{*} & \ell_{23}^{*} & \ell_{24}^{*}\\ \ell_{31}^{*} & \ell_{32}^{*} & \ell_{33}^{*} & \ell_{34}^{*}\\ \ell_{41}^{*} & \ell_{42}^{*} & \ell_{43}^{*} & 0 \end{array}\right]}}\overset{X}{\overset{︷}{\left[\begin{array}{cccc} X_{1}\left(t_{1}\right) & X_{1}\left(t_{2}\right) & X_{1}\left(t_{3}\right) & \cdots \\ X_{2}\left(t_{1}\right) & X_{2}\left(t_{2}\right) & X_{2}\left(t_{3}\right) & \cdots \\ X_{3}\left(t_{1}\right) & X_{3}\left(t_{2}\right) & X_{3}\left(t_{3}\right) & \cdots \\ j/F & j/F & j/F & \cdots \end{array}\right]}}. \end{array}$$

It follows that we can write Equation (A14) as the matrix equation:

(A18) $$\begin{array}{cc} c & =c^{0}+B^{c}\ell^{*}X\Delta t+B^{V}\ell^{*}X, \end{array}$$

where we now wish to vectorise the matrices such that the coefficient matrix is obtained as a vector that can be solved for using linear least squares regression. We fit transport coefficients to two different models, the ideal ion-selective model and the relaxed model, where the difference lies in the constraints on the $\ell^{*}$ matrix. In the ideal ion-selective model, there are four independent transport coefficients, $p_{i}$, that describe the $\ell^{*}$ matrix:

(A19) $$\begin{array}{c} \ell^{*,\mathrm{ideal}}=\overset{\ell^{c}}{\overset{︷}{\left[\begin{array}{cccc} 0 & 0 & 0 & 0\\ 0 & 0 & 0 & \frac{1}{2}\\ 0 & 0 & 0 & 0\\ 0 & -\frac{1}{2} & 0 & 0 \end{array}\right]}}+\overset{\ell^{p}}{\overset{︷}{\left[\begin{array}{cccc} 4p_{1} & -2p_{1} & 0 & p_{3}\\ -2p_{1} & p_{1} & 0 & -\frac{1}{2}p_{3}\\ 0 & 0 & p_{2} & p_{4}\\ -p_{3} & \frac{1}{2}p_{3} & -p_{4} & 0 \end{array}\right]}} \end{array}$$

For the relaxed model, it was still found necessary to set one element to zero, and the $\ell_{23}^{*}$ element was chosen, and we are left with 8 independent elements. The $\ell^{*}$ matrix then reads

(A20) $$\begin{array}{c} \ell^{*,\mathrm{relaxed}}=\left[\begin{array}{cccc} p_{1} & p_{2} & p_{4} & p_{6}\\ p_{2} & p_{3} & 0 & p_{7}\\ p_{4} & 0 & p_{5} & p_{8}\\ -p_{6} & -p_{7} & -p_{8} & 0 \end{array}\right]. \end{array}$$

In the relaxed model $\ell^{p,\mathrm{relaxed}}=\ell^{*,\mathrm{relaxed}}$ and $\ell^{c,\mathrm{relaxed}}=0$. The next step is to vectorise the matrix equation to redistribute the transport coefficients in a column vector. We use the vectorisation identity for matrices: $\mathrm{vec}\left(ABC\right)=\left(C^{T}⊗A\right)\mathrm{vec}\left(B\right)$, where ⊗ is the Kronecker product.

(A21) $$\begin{array}{c} \mathrm{vec}\left(c\right)=\mathrm{vec}\left(c^{0}\right)+\left[{\left(X\Delta t\right)}^{T}⊗B^{c}+X⊗B^{V}\right]\left(\mathrm{vec}\left(\ell^{p}\right)+\mathrm{vec}\left(\ell^{c}\right)\right). \end{array}$$

The vectorised parameter-dependent $\ell^{p}$-matrix can be written for the two different models as

(A22) $$\begin{array}{c} \mathrm{vec}\left(\ell^{p,\mathrm{ideal}}\right)=B^{p,\mathrm{ideal}}p^{\mathrm{ideal}}, \end{array}$$

where $p^{\mathrm{ideal}}={\left[p_{1},p_{2},p_{3},p_{4}\right]}^{T}$ and the $B^{p,\mathrm{ideal}}$ matrix is defined by the equation above. For the vectorised relaxed model matrix, we have

(A23) $$\begin{array}{c} \mathrm{vec}\left(\ell^{p,\mathrm{relaxed}}\right)=B^{p,\mathrm{relaxed}}p^{\mathrm{relaxed}}, \end{array}$$

where $p^{\mathrm{relaxed}}={\left[p_{1},p_{2},p_{3},p_{4},p_{5},p_{6},p_{7},p_{8}\right]}^{T}$ and the $B^{p,\mathrm{ideal}}$ matrix is defined by the equation above. Thus we can write

(A24) $$\begin{array}{c} \mathrm{vec}\left(c\right)=\mathrm{vec}\left(c^{0}\right)+\left[{\left(X\Delta t\right)}^{T}⊗B^{c}+X⊗B^{V}\right]\left(B^{p}p+\mathrm{vec}\left(\ell^{c}\right)\right). \end{array}$$

There are now two different options. We can choose to let the initial concentration be a fitting parameter, or we can decide to set the initial concentrations based on the first measurement point in the data series. If we use a fixed initial concentration from the data series, then we can move all terms that are not dependent on *p* to one side

(A25) $$\begin{array}{c} \overset{y}{\overset{︷}{\mathrm{vec}\left(c\right)-\mathrm{vec}\left(c^{0}\right)-\left[{\left(X\Delta t\right)}^{T}⊗B^{c}+X⊗B^{V}\right]\mathrm{vec}\left(\ell^{c}\right)}}=\overset{A}{\overset{︷}{\left[{\left(X\Delta t\right)}^{T}⊗B^{c}+X⊗B^{V}\right]B^{p}}}\overset{\beta }{\overset{︷}{p}}. \end{array}$$

We now have a matrix equation in the standard form Aβ=y for which the least squares solution for β can be found as $\beta ={\left(A^{T}A\right)}^{-1}A^{T}y$. If we choose to include the initial parameters as fitting parameters, we need to express $\mathrm{vec}(c^{0})$ as a vector of just the four initial ion concentrations:

(A26) $$\begin{array}{c} \mathrm{vec}\left(c^{0}\right)=\left[\begin{array}{c} c_{{\mathrm{Na}}^{+}}^{0}\\ c_{{\mathrm{Mg}}^{2+}}^{0}\\ c_{{\mathrm{SO}}_{4}^{2-}}^{0}\\ c_{{\mathrm{Cl}}^{-}}^{0}\\ 0\\ \vdots \end{array}\right]=\left[\begin{array}{cccc} 1 & 0 & 0 & 0\\ 0 & 1 & 0 & 0\\ 0 & 0 & 1 & 0\\ 0 & 0 & 0 & 1\\ 0 & 0 & 0 & 0\\ \vdots & \vdots & \vdots & \vdots \end{array}\right]\left[\begin{array}{c} c_{{\mathrm{Na}}^{+}}^{0}\\ c_{{\mathrm{Mg}}^{2+}}^{0}\\ c_{{\mathrm{SO}}_{4}^{2-}}^{0}\\ c_{{\mathrm{Cl}}^{-}}^{0} \end{array}\right]. \end{array}$$

Further, the *p* and the $c^{p}$ can be put together

(A27) $$\begin{array}{c} \tilde{p}=\left[\begin{array}{c} p\\ c^{p} \end{array}\right]. \end{array}$$

We can define matrices to pick out the *p* and $c^{p}$ parameters

(A28) $$\begin{array}{c} p=S^{p}\tilde{p}, \end{array}$$

(A29) $$\begin{array}{c} c^{p}=S^{c}\tilde{p}, \end{array}$$

where the selection matrices $S^{p}$ and $S^{c}$ have to be adjusted for the shape of the parameter vector *p* for each of the models. The matrix equation then reads

(A30) $$\begin{array}{c} \mathrm{vec}\left(c\right)=S^{c}\tilde{p}+\left[{\left(X\Delta t\right)}^{T}⊗B^{c}+X⊗B^{V}\right]B^{p}S^{p}\tilde{p}+\left[{\left(X\Delta t\right)}^{T}⊗B^{c}+X⊗B^{V}\right]\mathrm{vec}\left(\ell^{c}\right). \end{array}$$

The last step in the numerical fitting scheme is to extend the model to be able to fit to both current-producing and open-circuit condition measurements simultaneously. The adjustments that have to be made are just to stack the matrices and the initial concentration parameters described above and to add another selection matrix in the case where the initial concentration is used as a fitting parameter:

(A31) $$\begin{array}{c} {\tilde{p}}^{\mathrm{OC}+\mathrm{CP}}=\left[\begin{array}{c} p\\ c^{p,\mathrm{OC}}\\ c^{p,\mathrm{CP}} \end{array}\right], \end{array}$$

(A32) $$\begin{array}{c} p=S^{p}\tilde{p}, \end{array}$$

(A33) $$\begin{array}{c} c^{p,\mathrm{OC}}=S^{c,\mathrm{OC}}\tilde{p}, \end{array}$$

(A34) $$\begin{array}{c} c^{p,\mathrm{CP}}=S^{c,\mathrm{CP}}\tilde{p}. \end{array}$$

The full matrix equation with variable initial concentration then reads

(A35) $$\begin{array}{c} \overset{y}{\overset{︷}{\left[\begin{array}{c} \mathrm{vec}{\left(c\right)}^{\mathrm{OC}}\\ \mathrm{vec}{\left(c\right)}^{\mathrm{PC}} \end{array}\right]-\left[\begin{array}{c} {\left(X^{\mathrm{OC}}\Delta t^{\mathrm{OC}}\right)}^{T}⊗B^{c}+X^{\mathrm{OC}}⊗B^{V}\\ {\left(X^{\mathrm{CP}}\Delta t^{\mathrm{CP}}\right)}^{T}⊗B^{c}+X^{\mathrm{CP}}⊗B^{V} \end{array}\right]\mathrm{vec}\left(\ell^{c}\right)}}= \end{array}$$

(A36) $$\begin{array}{c} \overset{A}{\overset{︷}{\left[\begin{array}{c} S^{c,\mathrm{OC}}\\ S^{c,\mathrm{CP}} \end{array}\right]{\tilde{p}}^{\mathrm{OC}+\mathrm{CP}}+\left[\begin{array}{c} {\left(X^{\mathrm{OC}}\Delta t^{\mathrm{OC}}\right)}^{T}⊗B^{c}+X^{\mathrm{OC}}⊗B^{V}\\ {\left(X^{\mathrm{CP}}\Delta t^{\mathrm{CP}}\right)}^{T}⊗B^{c}+X^{\mathrm{CP}}⊗B^{V} \end{array}\right]B^{p}S^{p}}}\overset{\beta }{\overset{︷}{{\tilde{p}}^{\mathrm{OC}+\;\mathrm{CP}}}}. \end{array}$$

This is now a standard matrix equation for ${\tilde{p}}^{\mathrm{OC}+\mathrm{CP}}$ that can be solved with the standard linear least squares method. If the initial concentration is fixed, then the equation reads

(A37) $$\begin{array}{c} \overset{y}{\overset{︷}{\left[\begin{array}{c} \mathrm{vec}{\left(c\right)}^{\mathrm{OC}}\\ \mathrm{vec}{\left(c\right)}^{\mathrm{PC}} \end{array}\right]-\left[\begin{array}{c} \mathrm{vec}{\left(c^{0}\right)}^{\mathrm{OC}}\\ \mathrm{vec}{\left(c^{0}\right)}^{\mathrm{PC}} \end{array}\right]-\left[\begin{array}{c} {\left(X^{\mathrm{OC}}\Delta t^{\mathrm{OC}}\right)}^{T}⊗B^{c}+X^{\mathrm{OC}}⊗B^{V}\\ {\left(X^{\mathrm{CP}}\Delta t^{\mathrm{CP}}\right)}^{T}⊗B^{c}+X^{\mathrm{CP}}⊗B^{V} \end{array}\right]\mathrm{vec}\left(\ell^{c}\right)}}=\overset{A}{\overset{︷}{\left[\begin{array}{c} {\left(X^{\mathrm{OC}}\Delta t^{\mathrm{OC}}\right)}^{T}⊗B^{c}+X^{\mathrm{OC}}⊗B^{V}\\ {\left(X^{\mathrm{CP}}\Delta t^{\mathrm{CP}}\right)}^{T}⊗B^{c}+X^{\mathrm{CP}}⊗B^{V} \end{array}\right]B^{p}S^{p}}}\overset{\beta }{\overset{︷}{{\tilde{p}}^{\mathrm{OC}+\;\mathrm{CP}}}}. \end{array}$$

Finally, we include a weighting of the measured quantities. Several choices of weighting could be inferred. We have chosen to use the average of each measurement series to define the weights, such that the weight for an ion concentration in the OC measurement series is $w_{i}^{\mathrm{OC}}=1/\mathrm{avrg}{\left(c_{i}^{\mathrm{OC}}\right)}^{2}$, and similarly, for the emf $w_{V}^{\mathrm{OC}}=1/\mathrm{avrg}{\left(V^{\mathrm{OC}}\right)}^{2}$

(A38) $$\begin{array}{c} W^{\mathrm{OC}}=\left[\begin{array}{ccccccccc} w_{{\mathrm{Na}}^{+}}^{\mathrm{OC}} & \; & & \; & & & & & \\ \; & \ddots & \; & & \; & & & & \\ \; & \; & w_{{\mathrm{Mg}}^{2+}}^{\mathrm{OC}} & \; & & \; & & & \\ \; & \; & & \ddots & & & & & \\ \; & \; & & \; & w_{{\mathrm{SO}}_{4}^{2-}}^{\mathrm{OC}} & & & & \\ \; & \; & & \; & & \ddots & & & \\ \; & \; & & \; & & \; & w_{{\mathrm{Cl}}^{-}}^{\mathrm{OC}} & & \\ \; & \; & & \; & & \; & & \ddots & \\ \; & \; & & \; & & \; & & \; & w_{V}^{\mathrm{OC}} \end{array}\right]. \end{array}$$

The total weight matrix when fitting to open-circuit and current-producing experiments simultaneously is

(A39) WOC+CP=WOC WCP.

We additionally note that for some time steps, some of the concentrations were missing measurements. To avoid having to disregard the time step, we impute the data for these points. This is done by fitting coefficients $b_{0},b_{1}$ and $b_{2}$ for each measurement series to the assumed approximate time evolution

(A40) $$\begin{array}{c} c_{i}\left(t\right)=b_{0}+b_{1}\sqrt{t}+b_{2}t. \end{array}$$

The missing values were thereafter filled in using this formula. These imputed data points were used to calculate the driving forces. However, to avoid weighting these data points, the weights of these measurements were set to zero.

The least squares problem is finally solved as follows:

(A41) $$\begin{array}{c} \beta ={\left(A^{T}WA\right)}^{-1}A^{T}Wy. \end{array}$$

The residuals, *r*, are then

(A42) $$\begin{array}{c} r=y-A\beta . \end{array}$$

The variance is calculated as follows:

(A43) $$\begin{array}{c} \sigma^{2}=\frac{r^{T}W^{\mathrm{OC}+\mathrm{CP}}r}{n_{y}-n_{\beta }}, \end{array}$$

where $n_{y}$ and $n_{\beta }$ are the lengths of the *y* and β vectors.
